# MPRAnalyze: statistical framework for massively parallel reporter assays

**DOI:** 10.1186/s13059-019-1787-z

**Published:** 2019-09-02

**Authors:** Tal Ashuach, David S. Fischer, Anat Kreimer, Nadav Ahituv, Fabian J. Theis, Nir Yosef

**Affiliations:** 1 Department of Electrical Engineering and Computer Sciences, University of California Berkeley, Berkeley, California USA; 2 Center for Computational Biology, University of California Berkeley, Berkeley, California USA; 3 Institute of Computational Biology, Helmholz Zentrum München, Neuherberg, Germany; 4 TUM School of Life Sciences Weihenstephan, Technical University of Munich, Freising, Germany; 5 Department of Bioengineering and Therapeutic Sciences, University of California San Francisco, San Francisco, California USA; 6 Institute for Human Genetics, University of California San Francisco, San Francisco, California USA; 7 Ragon Institute of MGH, MIT, and Harvard, Cambridge, MA USA; 8 Chan Zuckerberg BioHub, San Francisco, California USA

## Abstract

Massively parallel reporter assays (MPRAs) can measure the regulatory function of thousands of DNA sequences in a single experiment. Despite growing popularity, MPRA studies are limited by a lack of a unified framework for analyzing the resulting data. Here we present MPRAnalyze: a statistical framework for analyzing MPRA count data. Our model leverages the unique structure of MPRA data to quantify the function of regulatory sequences, compare sequences’ activity across different conditions, and provide necessary flexibility in an evolving field. We demonstrate the accuracy and applicability of MPRAnalyze on simulated and published data and compare it with existing methods.

**Electronic supplementary material:**

The online version of this article (10.1186/s13059-019-1787-z) contains supplementary material, which is available to authorized users.

## Background

Understanding the function of the non-coding genome poses one of the most significant and outstanding challenges following the completion of the human genome project [1]. One critical function that is primarily associated with non-coding regions is to regulate the transcription of nearby genes by interaction with transcription factors and other proteins and through recruitment of the RNA polymerase complex [2, 3]. Two of the main classes of regulatory regions consist of promoters (which are proximal to the transcription start site of the respective gene) and enhancers (distal elements), both demonstrated to harbor many disease-related mutations [4, 5]. The delineation of these critical regions on a genome-wide scale has traditionally relied on chromatin-associated features that are indicative of regulatory activity, such as acetylation or methylation of certain residues along histone tails [1]. However, this approach does not provide direct evidence for regulatory activity, nor the dependence of this putative activity on the cellular context or on the presence of mutations.

Recent advances in reporter assays address this issue in a set of procedures dubbed massively parallel reporter assays (MPRAs) [6, 7]. In these assays, a synthetic DNA construct that contains a minimal transcriptional unit is introduced into cells. Each such construct is generally composed of a candidate regulatory sequence of interest, a minimal promoter, and a unique DNA “barcode” that can be transcribed. The candidate sequences are assumed to be capable of regulating the transcription of the barcode sequence similarly to how a native sequence may regulate the transcription of its target gene. The cells then undergo RNA and DNA sequencing to measure both RNA transcript counts and DNA construct counts, and the RNA-to-DNA ratio is used to estimate the transcription rate of every barcode. Relying on sequence-based reporters leverages the vast combinatorial space of unique sequences (instead of a limited set of fluorescent reporters [8]), and utilizes next-generation sequencing to measure the activity of thousands of putative regulatory sequences in a single experiment. To ensure robustness, each candidate regulatory sequence is usually associated with several barcodes (<10 to over 100, depending on the study).

MPRAs can be used to address several important questions. In classification studies, MPRAs are used to identify which putative regulatory regions are indeed inducing transcription (albeit in a synthetic context) [9, 10]. In allelic comparison studies, MPRAs are used to quantify the effect that variations to the sequence of regulatory regions may have on their ability to regulate transcription. This approach is primarily utilized for studying the effect of genetic polymorphisms that are observed in humans [11–13], but also to explore more basic science questions such as the effect of perturbing the sequence content, spacing, or number of transcription factor binding sites [13–15]. In comparative studies, MPRAs are used to quantify the dependence between the regulatory activity of each sequence and the cellular context, comparing tissues [16], cell lines [11], or other conditions of interest [17]. A combination of two or more study types is also possible through more complex experimental designs, for example measuring the interaction between alleles and conditions [12].

Despite growing popularity of MPRAs, most studies to date rely on analysis approaches that either discount the inherent noise in the data (e.g., taking an average ratio across all barcodes) or designed for other data modalities (such as DESeq2 [18], typically used for RNA-seq data, whose underlying assumptions may not hold true for MPRA). Other MPRA analysis methods only address some of the types of questions MPRAs can address, such as QuASAR-MPRA [19] and mpralm [20] that only perform comparative analyses, and rely on ratio-based summary statistics that limits the statistical power provided in these experiments. To address this, we have developed MPRAnalyze—a statistical framework that leverages information from multiple barcodes to ensure robust analysis of MPRA data. In the following, we demonstrate the use of MPRAnalyze for the three primary analysis tasks listed above, and compare its performance to the existing approaches using a collection of published datasets. MPRAnalyze is available as an R package through Bioconductor [21].

## Results

**Fig. 1:**
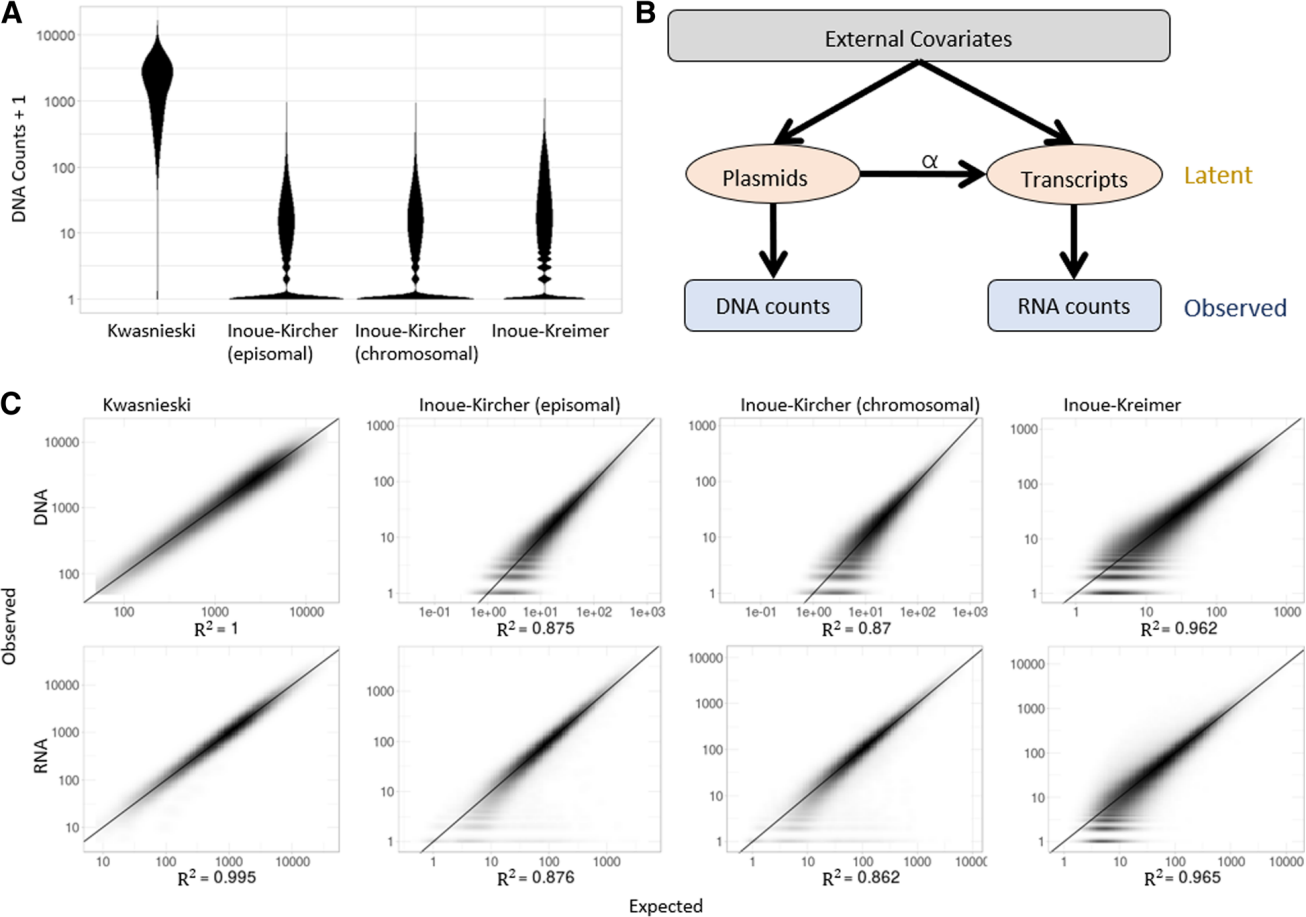
MPRAnalyze model properties and fit. **a** Distribution of construct abundances (DNA barcodes) across datasets, computed as the observed barcode count + 1 for visualization purposes. **b** A graphical representation of the MPRAnalyze model. External covariates (e.g., conditions of interest, batch effects, barcode effects) are design-dependent. Latent construct and transcript counts are related by the transcription rate *α*. **c** Goodness of fit plots for both DNA and RNA libraries across datasets. Expected counts were extracted from the fitted GLMs. MPRAnalyze’s model fits MPRA data well, with *R*^2^>0.86 across all datasets. Since the Kwasnieski data only has one replicate in the DNA library, the DNA model is able to reach a perfect fit, in which case the DNA estimates used in the RNA model are identical to the original DNA counts

MPRA data is produced from two parallel procedures: RNA-sequencing is used to measure the number of transcripts produced from each barcode, and DNA-sequencing is used to measure the number of construct copies of each barcode. Thus for each barcode the ratio of RNA to DNA can serve as a conceptual proxy for the transcription rate [7]. However, both DNA and RNA measurement procedures provide an approximate and noisy estimation, an issue exacerbated by the unstable nature of a ratio: minor differences in the counts themselves can result in major shifts in the ratio, especially when dealing with small numbers. This problem can be handled by associating multiple barcodes with each sequence, providing multiple replicates within a single experiment and a single sequencing library. This approach introduces an additional problem of summarizing counts from multiple barcodes to get a single transcription rate estimate for a candidate regulatory sequence, which is made difficult since the efficiency of incorporation inside cells, while theoretically uniform across the different constructs, has a significant degree of variability in practice (Fig. 1a). Two commonly used techniques of addressing this issue are based on summary statistics: the aggregated ratio, which is the ratio of the sum of RNA counts across barcodes divided by the sum of DNA counts across barcodes $\left (\frac {\frac {1}{n}\sum _{i}^{n} RNA_{i}}{\frac {1}{m}\sum _{j}^{m} DNA_{j}}\right)$; and the mean ratio, which is the mean of the observed RNA/DNA ratios across barcodes $\left (\frac {1}{n}\sum _{i}^{n}\frac {DNA_{i}}{RNA_{i}}\right)$. Although intuitive, both summary statistics have inherent limitations. The aggregated ratio loses the statistical power that multiple barcodes provide and is often dominated by a minority of barcodes with high counts, and the mean ratio is highly sensitive to noise, as recently demonstrated in a paper by Myint and colleagues [20]. A method to leverage the multiplicity of barcodes in a robust manner is therefore needed to fully fulfill the potential of these assays.

### MPRAnalyze model

We introduce MPRAnalyze, a method for the analysis of MPRA data that uses a graphical model to relate the DNA and RNA counts, account for the uncertainty in both libraries and leverage the unique structure and opportunities presented by MPRA data. Out model relies on the assumption of a linear relationship between the RNA counts and the corresponding DNA counts: *RNA*=*DNA*×*α*, similar to ratio-based approaches, with *α* denoting the transcription rate. Our framework comprises two nested models: the DNA model, which estimates the latent construct counts for the observed DNA counts, and the RNA model, which uses the construct count estimates from the DNA model and the observed RNA counts to estimates the rate of transcription, *α* (Fig. 1b).

For each candidate regulatory sequence, the model requires two vectors of observations: DNA counts $\vec {d}$ and RNA counts $\vec {r}$, where each observation is the number of times a specific barcode, associated with this sequence, was observed at the DNA and RNA levels respectively. Additionally, we denote $\vec {\hat {d}}$ the vector of latent construct counts (DNA) and $\vec {\hat {r}}$ the vector of latent transcript counts (RNA). We assume that the latent construct counts, from which the observed DNA counts are sampled, are generated by a gamma distribution. Second, we assume that the conditional distribution of the RNA counts follows a Poisson distribution. Formally:

1 $$ \vec{\hat{d}}\sim Gamma\left(k,b\right)  $$

2 $$ \vec{\hat{r}}|\vec{\hat{d}}\sim Poisson\left(\alpha\vec{\hat{d}}\right)  $$

These result in a closed-form negative binomial likelihood for the RNA counts:

3 $$ \vec{\hat{r}}\sim NB\left(\mu=\frac{\alpha\cdot k}{\beta},\ \psi=k\right)  $$

The negative binomial distribution is a common approximation of sequencing data due to the observed over-dispersion [22], and indeed all datasets we examined have a quadratic relationship between the mean and the variance, which can be captured by a negative binomial. This relationship is also observed for the DNA libraries, which is expected of Gamma-distributed data if the distribution’s shape parameter *k*≈1 (Additional file 1: Figures S1, S3; “Methods”).

Now, assume we have two conditions. In this case, each barcode is measured twice (once in each condition), and the model needs to relate these observations and account for potential differences between them. MPRAnalyze achieves this by assuming that the effects are log-additive, and replacing the simple components of the DNA estimate ($\vec {\hat {d}}$) and the transcription rate estimate (*α*) with generalized linear models (GLM) that enable easy encoding of various relationships between experimental factors. The model then becomes:

4 $$ \log\left(\vec{d}\right) = X_{D}\vec{\beta} + \log\left(\vec{S_{D}}\right)  $$

5 $$\log\left(\vec{r}\right) = X_{D}\vec{\beta} + X_{R}\vec{\gamma} + \log\left(\vec{S_{R}}\right)  $$

Here, *S*_*D*_,*S*_*R*_ are external correction factors, used to account for various technical effects such as library size in the DNA and RNA data respectively. *X*_*D*_,*X*_*R*_ are design matrices for the DNA and RNA models, which encode the experimental setup of the assay. For instance, in a two condition settings, each matrix will include a column with a 0/1 indicator corresponding to the first or second condition respectively. The respective coefficients *β* and *γ* will then capture the effect associated with the choice of condition. Notably, the DNA design matrix *X*_*D*_ will also usually encode the identity of the barcode, so as to enable per-barcode estimation of construct abundance. This is not necessary for the RNA design matrix *X*_*R*_ since we assume that the barcodes are replicates that should have a single estimate of the transcription rate. An illustrative example is provided in Figure S2 (Additional file 1) and a formal description of the model is provided in Additional file 2.

The model can be further extended to encode multiple covariates, both quantitative and qualitative, and thus support the common structure of MPRA experiments, namely multiple barcodes per sequence, multiple replicates or batches, and multiple conditions analyzed simultaneously. An important aspect of this flexibility is that it supports “un-paired” datasets in which the DNA sequencing was performed on the pool of constructs, prior to incorporation into cells [10–13]. In these cases, there might not be separate DNA estimates for each condition being tested, in which case the conditions of interest would only be modelled in the RNA design matrix and excluded from the DNA model.

In summary, MPRAnalyze utilizes a model that accounts for barcode specific effects and leverages them for increased statistical power and robustness of estimation. Since a standard for MPRA experimental design has yet to be formed, the nested GLM construction provides flexibility and is easily adjustable to changing experimental designs. Our model is also highly interpretable, allowing for quantitative estimates of sequence activity to be easily extracted, as well as differential activity to be tested directly using established statistical tests. This framework can explicitly leverage negative controls (sequences with no expected regulatory function) when available, either to establish the null distribution in classification analyses or to correct for systemic bias in comparative analyses (“Methods”).

### Benchmark datasets

In the following sections, we investigate the performance of MPRAnalyze in quantifying the transcriptional activity of candidate regions, as well as in the three major analysis tasks, namely—classification, cross-condition analysis, and allelic comparisons. Finally, we evaluate MPRAnalyze in a complex setup where we investigate both multiple conditions and multiple alleles. We compare MPRAnalyze to the current set of tools and analysis methodologies, using simulated data and a collection of public data sets. These datasets were chosen for representing a diversity of MPRA protocols (e.g., episomal or lentiviral integration, DNA sequencing pre- or post-transduction), study focus (classification, comparative analyses, allelic comparisons), and experimental design (number of barcodes per sequence, number of replicates). A summary of the data sets and their properties is provided in Table 1. Applying MPRAnalyze to these data, we found that the model is able to provide a good fit (*R*^2^>0.86 for all datasets, Fig. 1c), which is consistent with our distributional assumptions (Additional file 1: Figure S3).

**Table 1:** MPRA datasets used for evaluation of MPRAnalyze throughout the paper

Dataset	Type of analysis	Integration	DNA sequencing	#Sequences	#Negative controls	#Barcodes	#Replicates (DNA,RNA)
Kwasnieski [10]	Quantification	Episomal	Pre-transduction	1200	568	4	1,4
Inoue-Kircher (epi) [9]	Quantification	Episomal	Post-transduction	2338	102	100	3,3
Inoue-Kircher (chr) [9]	Quantification	Lentiviral	Post-transduction	2338	102	100	3,3
Inoue-Kreimer [17]	Comparative	Lentiviral	Post-transduction	2464	200	90	3,3
Mattioli [13]	Allelic Comparison	Episomal	Pre-transduction	3960	0	26/80	1, 4/8

In the Mattioli data, multiple values indicate an asymmetric design: reference alleles were associated with 80 barcodes compared with 26 barcodes for alternative alleles, and 4 replicates were available for K562 cells compared with 8 in HepG2. For further details on each datasets, see “Methods”

### Quantification

We set out to examine the properties of the estimate of transcription rate generated by MPRAnalyze, denoted *α* (alpha), and compare it to the ratio-based summary statistics (i.e., mean of RNA-to-DNA ratios across all barcodes, or alternatively, the ratio of means [henceforth referred to as the *aggregate ratio*]).

**Fig. 2:**
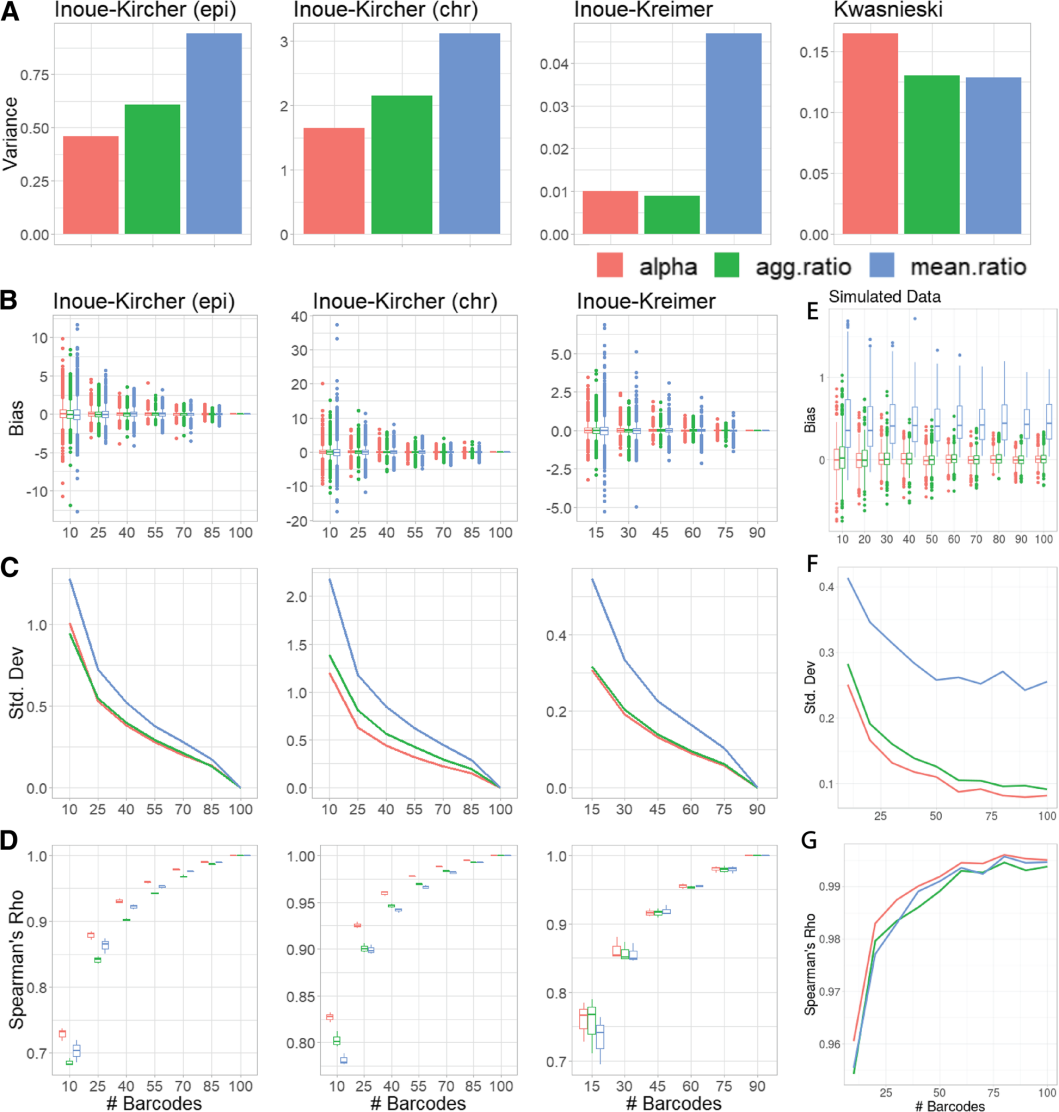
Comparison of MPRAnalyze’s *α* estimate of transcription rate with the ratio-based estimates $\left (\text {agg.ratio:} \frac {\frac {1}{n}\sum _{i}^{n} RNA_{i}}{\frac {1}{m}\sum _{j}^{m} DNA_{j}}; \text {mean.ratio:} \frac {1}{n}\sum _{i}^{n}\frac {DNA_{i}}{RNA_{i}}\right)$**a** The variance measured among estimates of negative-control sequences in each dataset (these are assumed to have an identical transcription rate). **b–d** Barcodes were sampled and quantification was recomputed based on the partial data to measure the effect of barcode number on estimate performance [See “Methods” for further subsampling details]. Analyses were performed using the full-data estimate as the ground truth. **e–g** MPRA data was simulated to provide an actual ground truth. In each case we measured the bias (*estimate*−*truth*) (**b,e**); the standard deviation $\left (\sqrt {Var\left (estimate-truth\right)}\right)$ (**c,f**); and the Spearman correlation between the estimates and the ground truth (**d,g**)

Reassuringly, the three estimates are largely in agreement (Pearson’s *r*>0.9 across datasets, Additional file 1: Figure S4). To further examine the accuracy of the estimates, we used the negative control sequences included in some of the datasets. These are assumed to have an identical transcription rate induced by the minimal promoter included in each construct with no sequence-induced activity. We examined the variance of the estimates on these sets. In the Kwasnieski dataset, the limited number of barcodes (*n*=4) is mitigated by high counts per barcode (Fig. 1a), leading to all estimates having similarly low variance. In the barcode-rich datasets (*n*≥90), the mean ratio is expectedly [20] the most variable, with *α* being the most consistent in the Inoue-Kircher datasets and comparably consistent to the aggregated ratio in the Inoue-Kreimer dataset (Fig. 2a). These results suggest that MPRAnalyze is estimating similar transcription rates across the negative controls, as expected from this collection.

We then explored the effect of the number of barcodes on the estimates’ performance. Using the barcode-rich datasets, barcodes were sampled at various rates and estimates were recomputed for each sequence (3 independent samples per sequence per barcode rate). Using the full-data estimates as the ground truth, we found that down-sampling barcodes does not result in a systemic bias in any of the estimates (Fig. 2b), and all estimates showed reduced variance with increased barcodes, with the mean ratio under-performing the other two estimates, and *α* having a similar or lower variance than the aggregated ratio (Fig. 2c).

In many cases, the goal of quantifying sequence activity is to rank and compare different sequences, as in mutagenesis experiments. To compare the stability of the ordering of sequences, the Spearman correlation was computed between the estimates in each sub-sample to the estimates of the full data. Alpha has either similar or higher correlation than both ratio-based estimates across datasets and barcode abundance (Fig. 2d).

Since these analyses are limited by a lack of ground truth, MPRA data was then simulated by generating random coefficients and using the same nested GLM construction as described above to generate samples. To avoid biasing the results, samples were generated with a log-normal noise model instead of the default Gamma-Poisson convolutional model MPRAnalyze uses (“Methods”). We generated 281 sequences with gradually increasing transcription rates spanning a range of possible values (from 0.2 to 3, in 0.01 steps), with three replicates in each simulation. The analyses above were repeated with the simulated data. We found that while the measured bias was indeed not influenced by the number of barcodes, the mean ratio is substantially more biased than both *α* and the aggregated ratio (Fig. 2e). Similar to the real data results, we found *α* has lower variance than both ratio-based estimates, and higher correlation with the true transcription rates (Fig. 2f, g). We also simulated data with varying number of replicates and found that increasing the number of replicates has a similar effect to increasing the number of barcodes, since both parameters increase the effective sample size. With any given number of barcodes, increasing the number of replicates improved performance—the degree of improvement decreased when more barcodes were available (Additional file 1: Figure S5).

Overall, we found that *α* performs similarly or better than both ratio-based estimators in terms of accuracy, consistency, and robustness to missing data.

### Classification

A common use case for MPRA is classification of active sequences, which induce transcriptional activity. This is commonly done by comparing the ratio-based estimates of the assayed sequences to a control set of sequences [9, 10], an approach that suffers from the summary statistics’ sensitivity to noise and missing data, demonstrated above, which in the context of classification leads to decreased power and accuracy. Other studies performed this analysis using DESeq2 [18], a differential expression analysis (DEA) method, by treating the DNA and RNA libraries as two conditions and looking for significant differences between the two [11]. In the following we demonstrate that overall DEA methods either lack power or are not well calibrated for MPRA data. More importantly, these methods rely on an implicit assumption that the majority of features do not display differential behavior, a valid assumption for RNA-seq that does not hold for MPRA, in which the assayed sequences are often explicitly selected for their potential activity. This assumption makes the results of DEA methods highly dependent on experimental design and sequence selection.

MPRAnalyze performs classification of active sequences by comparing the respective *α* estimates against the null distribution of transcription rate induced solely by the minimal promoter. The null is based on negative control sequences when available, and otherwise, MPRAnalyze relies on a conservative assumption that the mode of the distribution of the *α* values is the mode of the null distribution and that values lower than the mode are broadly generated by the null. These values are therefore used to estimate the mean and variance of the null distribution.

In both scenarios, the *α* value of each candidate sequence is compared against the null distribution using the median absolute deviation (MAD)—a variant of the *Z*-score that is less sensitive to outliers. MPRAnalyze supports either a one-sided or two-sided test, allowing for identification of inducing sequences (inducing transcription beyond the minimal promoter levels) or repressive sequences (repressing transcription to below the promoter levels). A one-sided test was used to generate all results presented in this paper.

#### Comparing mPRAnalyze with existing methods

To assess the performance of MPRAnalyze in classification analyses, we compared six methods: MPRAnalyze with and without negative controls; empirical *p*values computed using the two ratio-based estimates, and DESeq2 in either full mode (each barcode as a separate sample) or collapsed mode (each replicate as a sample, taking the sum across barcodes within each replicate; see “Methods”). Similarly to MPRAnalyze, DESeq2 was applied using an asymmetric mode, namely focusing on inducing sequences that have a higher signal in the RNA library than in the DNA library.

**Fig. 3:**
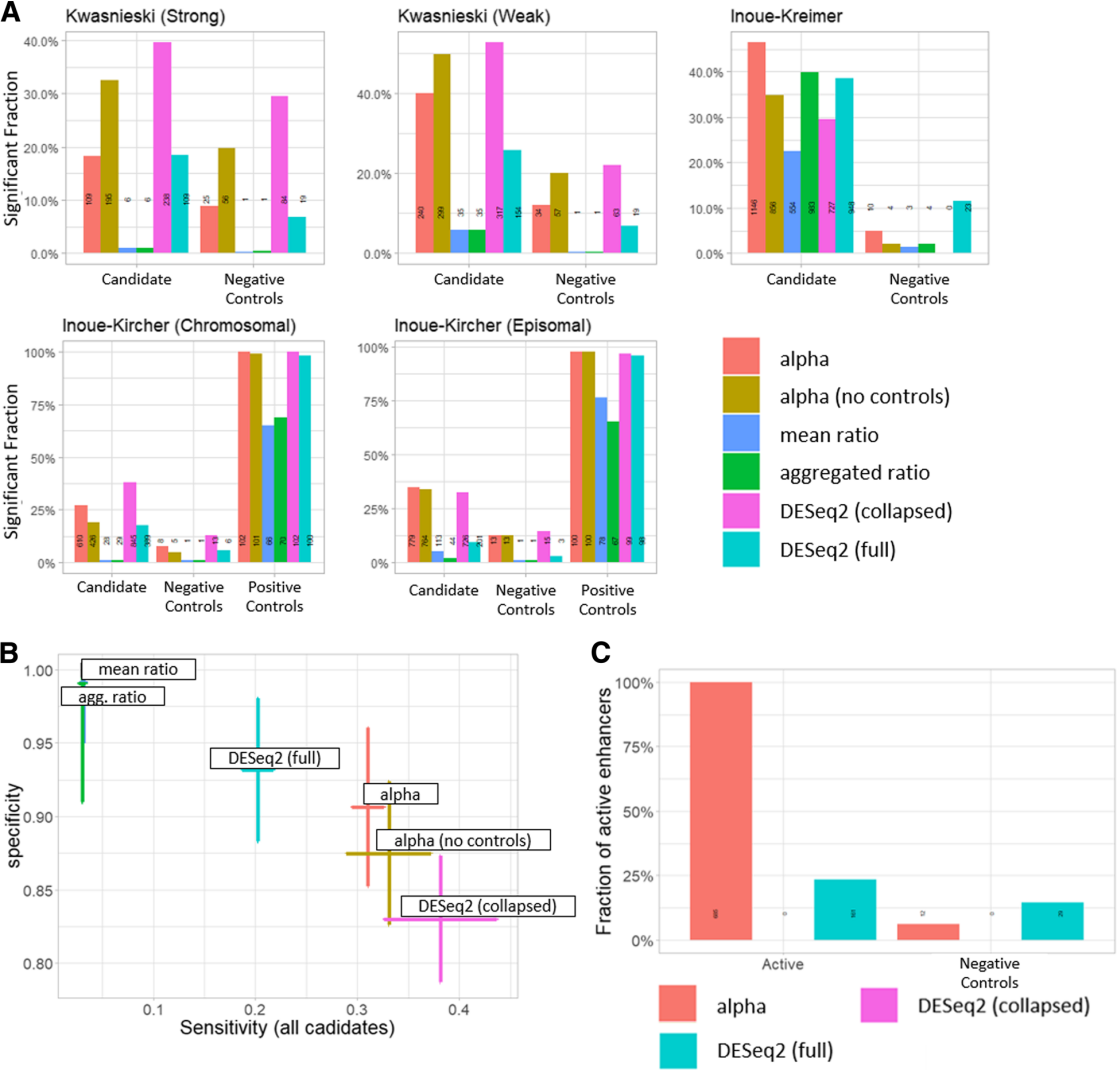
Classification analysis comparisons. **a** fraction of sequences identified as significantly active (BH-corrected *P*<0.05) by method and class of sequence. MPRAnalyze results both in control-based (red) and no-controls (orange) modes; empirical *p* values based on the mean ratio (blue) or aggregated ratio (green); DESeq2 results in collapsed mode (barcodes are summed within each batch, purple) or full mode (full data, light blue). Absolute number of active sequences is displayed on the bars. **b** Precision-Recall curve. Precision is based on performance on the negative controls, Recall is based on the total population of sequences, assuming all candidates are active. Error bars are ± the standard deviation of these measures across datasets. **c** Fraction of active sequences detected after re-running the analyses on 685 sequences from the Inoue-Kreimer dataset that were identified as active by MPRAnalyze (regular mode) and both DESeq2 modes, and the 200 controls from the same dataset. MPRAnalyze recapitulates the same results, finding that 100% of the candidates are active, whereas DESeq2 full only identifies 161 (23.5%) and DESeq2 collapsed completely fails to identify any active sequences

We examined the fraction of sequences that were significantly active (FDR <0.05) in each dataset, stratified by group: negative controls, candidate sequences, and positive controls when available (Fig. 3a). As expected, empirical *p* values from the ratio-based estimates show a clear lack of power. Both DESeq2-collapsed and MPRAnalyze without controls have inflated rates of false positives in the Kwasnieski datasets (compared with the theoretically expected 5% false discovery rate among the negative controls set). When examining the results across all datasets, we find that while MPRAnalyze and DESeq2 have overall comparable results, both modes of MPRAnalyze achieve a better balance between sensitivity (identifying candidates as active) and specificity (not identifying negative controls as active) than both modes of DESeq2 (Fig. 3b).

Since the above analysis overlooks the overall statistical behavior of the methods, we examined the full *p* value distribution of each method within each dataset. Considering multiple datasets, we found that both modes of MPRAnalyze, both ratio-based methods and DESeq2-full appear well calibrated, whereas DESeq2-collapsed does not follow the theoretical distribution of *p* values: a mixture of uniform values (corresponding to non-active sequences that follow the null distribution) and low values (active sequences for which the null is rejected) (Additional file 1: Figure S6). Similar results were found when examining the distribution over negative controls only (expected to be uniform), with MPRAnalyze in the no-control mode having some inflated values (assigning more low *p* values than expected), which emphasizes the importance of using negative controls in classification studies (Additional file 1: Figure S7). Finally, we examined the distribution over positive controls (only available in the Inoue-Kircher datasets) and found that MPRAnalyze in both modes has significantly higher statistical power, being outperformed only by ill-calibrated DESEq2-collapsed (Additional file 1: Figure S8). Overall, we found that despite comparable rates of sequences found statistically significant, the MPRAnalyze model is better calibrated to MPRA data.

#### Caveats of using methods designed for differential expression

DESeq2 pools information across all features included in the dataset (genes for RNA-seq, candidate enhancers for MPRA), both in the library size correction and estimation of the dispersion parameter. However, unlike genome-wide assays such as RNA-seq, the set of assayed features in MPRA experiments is curated according to the specific goals and context of the study. We hypothesized that DESeq2-based classification would be highly dependent on the sequences included in the analysis. We repeated the classification analysis on the Inoue-Kreimer dataset using only the 200 negative controls sequences and 685 candidate sequences that were previously classified as active by MPRAnalyze and both modes of DESeq2. This simulated a scenario in which the data was generated in an experiment that included fewer sequences. Confirming our hypothesis, MPRAnalyze results remain unchanged with all candidate sequences significantly active, whereas DESeq2-full only classifies 161 (23.5%) of the sequences as active and DESeq2-collapsed finds no active sequences at all. This reveal an inherent limitation of using differential expression methods such as DESEq2 for analyzing MPRA data.

### Comparative studies

Another common use for MPRAs is comparative studies, looking for differential transcription induced by a putative regulatory sequence between different cell types, stimuli, or other experimental covariates [11, 16]. More complex experimental settings are also possible, e.g., using MPRA to evaluate transcriptional activity over time as in the Inoue-Kreimer data [17], or the interaction between differential allele activity and the presence of a certain transcription factor, as performed by Ulirsch and colleagues [12].

Here we use the Inoue-Kircher data to demonstrate that MPRAnalyze is more statistically powerful than establish methods for analyzing comparative MPRA data, and therefore enables discovery of more neuanced biological signals, and that MPRAnalyze supports more complex experimental designs that are not supported by previous methods (e.g., temporal analysis).

Performing differential activity analysis in MPRAnalyze can be done in two ways: first, since MPRAnlyze optimizes the model using likelihood maximization, any single hypothesis that can be encoded in a generalized linear model can be tested using a likelihood ratio test. This includes complex hypotheses that can be captured by interaction terms between covariates (e.g., cell type and genetic background [12]). Additionally, in simple two-condition designs, or in cases where multiple contrasts are compared to a single reference (e.g., multiple different stimuli compared against the unstimulated behavior), the model coefficients can be extracted from the RNA model and tested using a Wald test. While both options are supported in the implementation of MPRAnalyze, the results in this paper are based on likelihood ratio testing.

When performing comparative analysis, it is important to account for possible biases, such as those induced by overall differences in the basal transcription rate. In RNA-seq experiments, this issue is usually resolved via library size correction [23], but with MPRA this is not necessarily sufficient. This is because for the library size to properly correspond to bias in the data, either the vast majority of features must be non-differential, or the differential signal must be symmetric. Neither of these assumptions necessarily hold for MPRA data, as they largely depend on the selection of the candidate sequences. For instance, MPRA can be designed with most sequences being more active in one condition than in the other, and thus most sequences are indeed differentially active. To address this issue, MPRAnalyze utilizes negative controls in the data to define the null differential behavior. This is done by fitting a separate, joint model for the controls, in which each control sequence has a distinct DNA model but they all share a single RNA model, reflecting the basal activity in each condition (Methods).

Alternative methods have been developed to address this or similar questions. QuASAR-MPRA [19] was designed specifically for allelic comparisons and uses a beta-binomial model and mpralm [20] which is a general differential-activity tool designed for MPRA which fits a linear model. Both methods use summary statistics and do not include barcode-level information in their model. Mpralm can use either the aggregated ratio or the mean ratio as the statistic, and is therefore subject to the limitations described above. QuASAR-MPRA, similar to MPRAnalyze, models the DNA and RNA separately, but it does so using the sum of counts across all barcodes in each condition, collapsing the data into a single measurement.

#### Comparing mPRAnalyze with existing methods

**Fig. 4:**
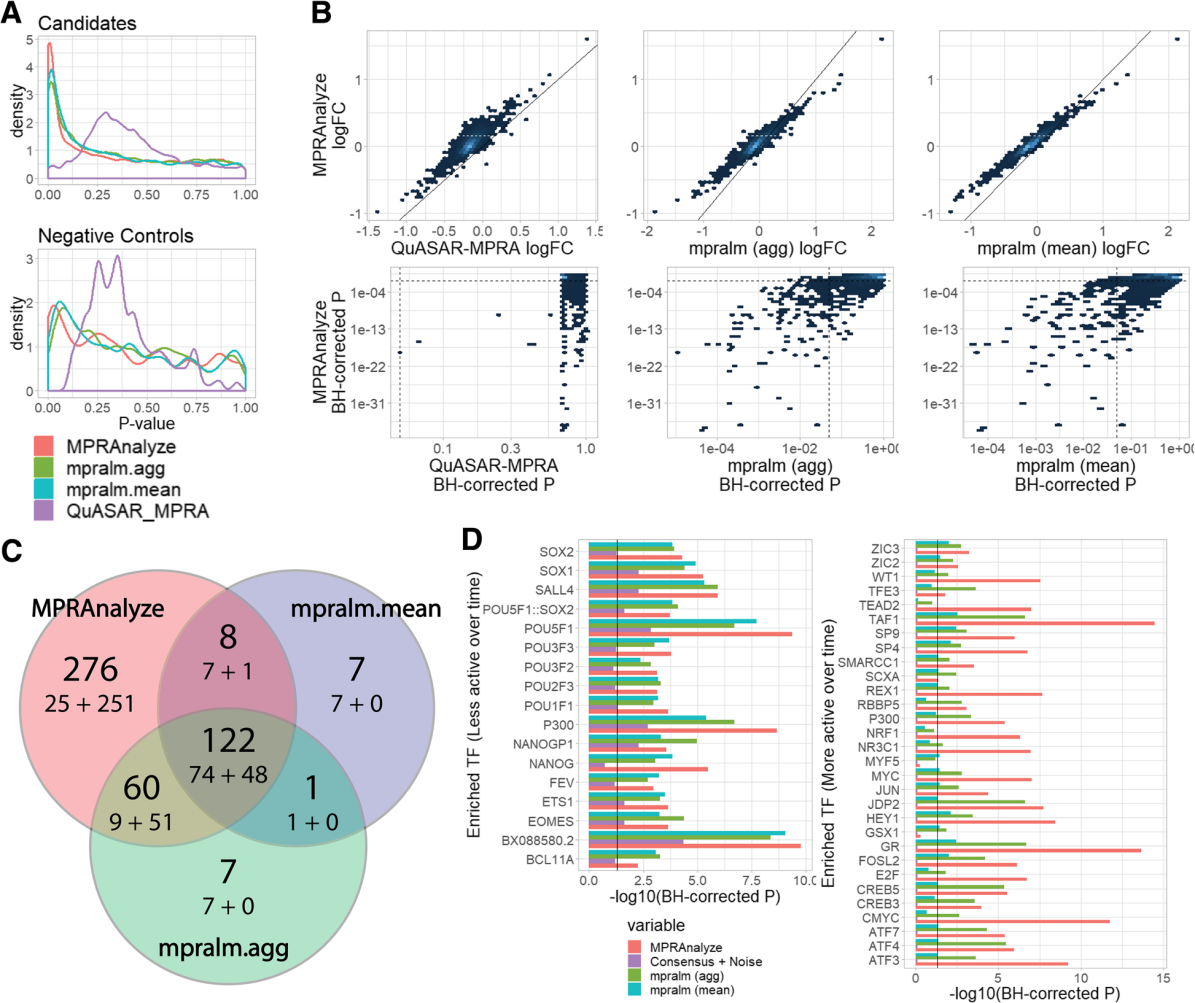
Comparative analysis results of comparing timepoint 0h to 72h in the Inoue-Kreimer dataset. **a***p* value distributions of candidates (top) and controls (bottom). QuASAR-MPRA is poorly calibrated, whereas MPRAnalyze and both mpralm modes follow the theoretical behavior (mixture of uniform and low values). **b** Direct comparison of MPRAnalyze to competing methods. Top panels show the biological effect size (log fold-change); Bottom panels show the statistical significance (BH-corrected *p*; dotted lines are 0.05 threshold). **c** Venn diagram for MPRAnalyze and mpralm (both modes). The numbers in each area are (top) the total number of sequences in the area, and (bottom) the number of decreasing-activity sequences (left) + and increasing-activity sequences (right). **d** Enrichment of transcription factor binding sites in differentially active sequences as determined by each method. Solid line represents threshold of 0.05. (see “Methods” for further details)

To compare these different methods, we used the Inoue-Kreimer dataset and extended the subset of samples we used to include both the 0 h and 72 h timepoints (post neural induction of human embryonic stem cells (hESC)). We then looked for sequences whose activity differed between the two time points, using MPRAnalyze, mpralm (both aggregated ratio and mean ratio modes), and QuASAR-MPRA (“Methods”). The distribution of *p* values (Fig. 4a) shows that overall MPRAnalyze and both modes of mpralm are well calibrated, following the expected mixture of uniform values and low values among candidates, and showing slight inflation but overall uniform behavior among the negative controls. Conversely, QuASAR-MPRA is less calibrated on both candidates and negative control sequences, recapitulating the results described by Myint et al. [20]. Indeed, QuASAR-MPRA only identified two candidates as significantly differential (BH-corrected *p* values <0.05).

Overall, we observe that the estimates of effect size (log fold-change) are largely reproducible across methods (Pearson’s *r* >0.84 across all pairs). In terms of statistical power (Fig. 4b), we observe that MPRAnalyze calls more sequences as significant compared to the other methods. We further note that the FDR values of MPRAnalyze are largely correlated with those of mpralm among statistically significant candidates (Spearman correlation >0.63 for sequences MPRAnalyze calls differential) and that the estimates of QuASAR-MPRA do not correlate with the other two methods (consistent with the results in Fig. 4a). Further examination of the results excluded QuASAR-MPRA since it did not identify a sufficient number differential sequences.

We further examined the differential sequences, after filtering the results to only include candidate sequences that are classified as active in at least one of the conditions (BH-corrected *p*<0.05, using MPRAnalyze’s classification method). Interestingly, mpralm in aggregate mode finds a roughly balanced number of sequences that are increasing (99) and decreasing (91) in activity (comparing 0 h to 72 h), and in mean mode finds more decreasing (89) than increasing (49), while MPRAnalyze finds far more increasing (351) than decreasing (115) sequences (Fig. 4c). However, sequences in the Inoue-Kreimer study were explicitly selected to correspond to increased activity over the course of differentiation (2037 [82%] of the assayed sequences are genomic regions selected due to their closest gene showing increased expression over differentiation). Therefore, the imbalance in the results from MPRAnalyze fits to the design of the experiment.

We then explored the set of candidates that were detected by each method. To this end, we divided the set of differentially active sequences into decreasing and increasing activity (comparing 0 h to the 72 h time point), then within each set we tested for over-representation of DNA binding motifs (hypergeometric test, BH-corrected *p*<0.05; “Methods”). To narrow down the results, we examined the union of top 15 most enriched transcription factor binding motifs by each method (Fig. 4d, Additional file 1: Figure S9, Additional file 3: Table S1, Additional file 4: Table S2).

Among decreasing-activity sequences, we find as expected binding sites for two of the core pluripotent factors (NANOG, POU5F1). While these are captured by all methods, we observe a higher significance with MPRAnalyze. Among increasing-activity sequences, where the methods have more profound differences, we find that MPRAnalyze generally has lower fold-enrichment scores, but compensates by a substantial increase in statistical power. Overall, mpralm in *mean* mode does not detect many of the enriched transcription factors found by the other methods, with a total of 23 (compared with 106 and 195 found by mpralm *aggregate* and MPRAnalyze, respectively), and displays diminished statistical power.

To ensure that these results are not simply explained by the higher number of differential sequences detected by MPRAnalyze, we also examined a *consensus + noise* option, where the consensus set (sequences called differential by all methods) was inflated with randomly chosen sequences (taken from the remaining population) to match the number of differential sequences called by MPRAnalyze (“Methods”). We find that this simulated inflation that does not reflect true biological signal does not explain the increased power displayed by MPRAnalyze.

Notably, MPRAnalyze results are enriched for binding sites for TEAD2 and NRF1, but results accourding to the other methods do not contain such enrichment. Both factors have been implicated in neurogenesis by previous studies [24, 25], and upon closer examination we found that NRF1 binding sites have comparable fold-enrichment in all methods (1.48 in MPRAnalyze, 1.39 in mpralm *aggregate* and 1.45 in mpralm *mean*), but only pass the statistical threshold with MPRAnalyze. In the other direction, we found the mpralm results are enriched for binding sites of MYF5 and GSX1, but not the MPRAnalyze results. However, when examining the mRNA levels measured in the corresponding time points, we found that both factors have very low expression levels in the conditions in which MPRA was conducted (Additional file 5: Table S3). These levels are below their characteristic expression levels in tissues they are known to be active in [26], making them less attractive candidates for driving differential transcription. Overall, MPRAnalyze identifies biological signal that is consistent with the competing methods, with increased statistical power, which allows for more nuanced results.

#### Detecting temporal activity

Finally, we note that MPRAnalyze can be used on the entire Inoue-Kreimer dataset, which consists of seven time-points, to identify sequences whose activity changes over time. MPRAnalyze performs this analysis by comparing two models: the full model, which allows for time-dependent activity; and the reduced model, in which time-point factors are excluded, thereby forcing a constant behavior across time-points (methods). This analysis cannot be performed by either of the competing methods: QuASAR-MPRA only supports two-condition comparisons, and mpralm only supports coefficient-based hypothesis testing. We ran MPRAnalyze in this fashion and after filtering sequences to only those that are active in at least one time-point (FDR <0.05, using MPRAnalyze to perform classification analysis per time-point) MPRAnalyze finds 749 (28%) sequences that have temporal activity (methods, FDR <0.05). Reassuringly, of the 466 sequences identified as differential between the first and last time-points, 420 (90.1%) are found to have overall temporal activity.

We found that temporal sequences broadly tend to have a smooth impulse-like activation pattern over time [27], whereas negative control sequences have less clear patterns (Additional file 1: Figure S10). We then clustered the temporal sequences (*K*-means with *K* = 4 on *α* values, *z*-normalized for each sequence) in order to group sequences with a similar temporal behavior pattern, and repeated the same binding site enrichment analysis as above (Additional file 6: Table S4) for each cluster. As evidence for the validity of our approach, we found that the sequences that are active at the early time points were indeed enriched for binding sites of core pluripotent factors (NANOG, SOX2, POUF51), and that sequences that are active later in the differentiation process were enriched for binding sites of transcription factors known to participate in neural differentiation (ATF2 [28], HES1 [29], GLI1, LEF [30]).

### Allelic comparison

Many MPRA studies deal with quantifying the effect of sequence variants on regulatory function. These studies, referred to here as allelic comparison studies, include those that compare observed genetic variants to investigate the regulatory effect of different alleles of a regulatory sequence [12], as well as studies that deliberately change a sequence to elucidate the regulatory grammar in a systemic fashion [13]. While conceptually similar to comparative analyses, allelic comparisons require different factors to be considered. Two important differences are: (1) the compared sequences (e.g., wild type and mutant allele) come from the same sample and therefore a systemic bias is less concerning than it is when comparing different conditions, and (2) the different alleles being compared are associated with different barcodes, in contrast with condition-wise comparison in which barcodes are shared between conditions.

To demonstrate the utility of MPRAnalyze in this scenario, we used recently published data by Mattioli and colleagues [13], who measured the effects of all possible single-nucleotide deletions were examined on 31 selected promoters. To this end, an MPRA was conducted with all the deletion and corresponding wild type (WT) sequences, where each deletion was associated with 26 barcodes and each WT sequence was associated with 80 barcodes. A single sample of the pre-transduction plasmids was sequenced to produce the DNA library. The RNA samples were taken from two different tissues: eight samples from the HepG2 cell line and four samples from the K562 cell line. This asymmetrical experimental design exemplifies the diverse nature of MPRA studies, and the necessity of a flexible framework.

Using this dataset, we demonstrate that MPRAnalyze is well calibrated and more statistically powerful than established methods, and supports studying the interaction of multiple conditions: in this case finding sequence variants with cell-line specific functional effects.

#### Comparing mPRAnalyze with existing methods

Similar to the comparative analysis described above, we compared each deletion sequence with the corresponding WT in each tissue separately, with all three methods: MPRAnalyze, mpralm (which only supports the *aggregated* mode for allelic comparisons), and QuASAR-MPRA.

**Fig. 5:**
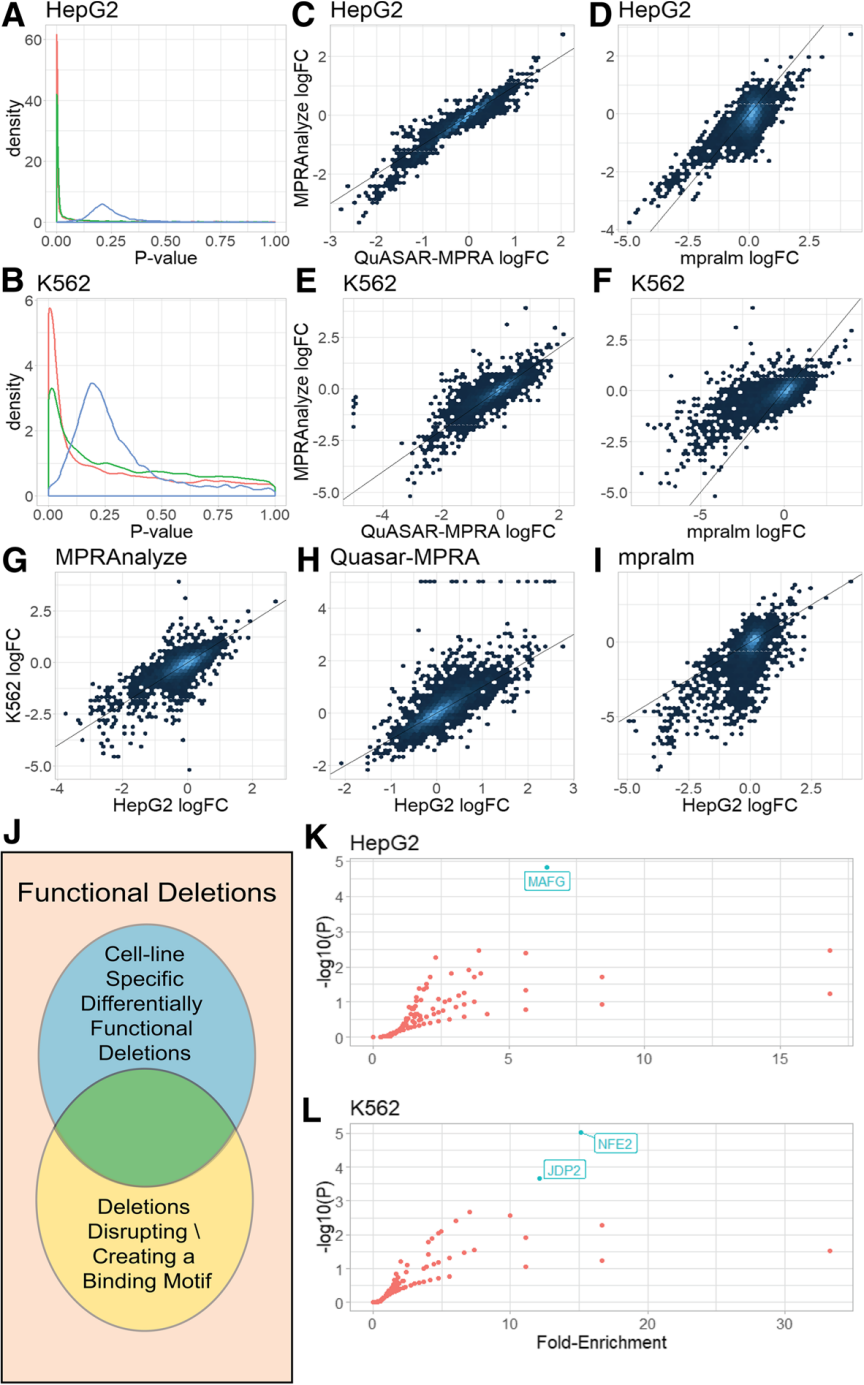
Performance evaluation in allelic comparison. **a,b***p* value density of the three evaluated methods in both cell lines. **c–f** logFC values between methods in each cell type shows all methods find a similar biological signal. **g–i** logFC values between cell types for each method. Some differences are expected, but overall values are highly correlated. **j** Schematic of the enrichment analysis, testing cell-line specific functional deletions for enrichment of motifs that were gained or lost by those deletions. **k, l** results of motif enrichment analyses. Transcription Factors with significant enrichment (FDR < 0.05) are labeled

When examining the *p* value distribution generated by each method we find that MPRAnalyze and mpralm are both better calibrated than QuASAR-MPRA (Fig. 5a-b). Consistent with our previous results, all methods have correlated estimates of biological effects (Fig. 5c–f). The methods are better correlated in the HepG2 data compared with the K562 data (correlations with MPRAnalyze: Pearson’s *r*=0.72 in K562 and 0.77 in HepG2 for mpralm, and 0.78 in K562 and 0.96 in HepG2 for QuASAR), which we hypothesized is due to the higher number of replicates in the HepG2 data. When the comparison was repeated using only four replicates of the HepG2 data, the correlations between methods decreased (correlations with MPRAnalyze: Pearson’s *r*=0.63 for mpralm and 0.38 for QuASAR, Additional file 1: Figure S11).

We then compared the effects estimated by each method across cell lines. Overall, we find a high degree of similarity in the effects of sequence perturbation across cell lines—a finding supported by all the methods we considered (Fig. 5g–i). Looking more closely, we find that mpralm and QuASAR-MPRA both find a systemic skew towards stronger effects in K562, with 72.6% and 63.1% of deletions having a more extreme log fold-change value in K562 compared with HepG2 in mpralm and QuASAR-MPRA, respectively, whereas MPRAnalyze results are more balanced, with 49.8%. When comparing statistical power, we again find that MPRAnalyze can detect more deletions that significantly affect the rate of transcription (FDR <0.05). In HepG2, MPRAnalyze finds 2855 (72%) deletions with a significant effect, whereas mpralm finds 2710 (68.4%), with 2071 (52.2%) of the sequences significant in both; in K562, MPRAnalyze finds 1230 (31%) significant deletions compared with 360 (9%) found by mpralm, with 272 (6.8%) significant in both. In both cell types, QuASAR-MPRA does not find any significantly functional deletion. As expected, due to the larger sample size, both MPRAnalyze and mpralm are more powerful in HepG2 compared with K562.

#### Identifying variants with cell-line specific effects

Since the Mattioli study performed allelic comparisons in two cell types, it can also be used for the identification of deletions that have a different effect in HepG2 cells compared with K562 cells. With MPRAnalyze, it is possible to address this question directly, testing the interaction between the tissue and the allele covariates in the model. When performing this analysis, MPRAnalyze found 608 (15.3%) differential deletions that had a different effect between cell types. For example, the core promoter of the lncRNA gene DLEU1 has several functional deletions that are highly concordant between cell types, and a single differentially functional deletion in position 83, where the deletion has a significantly larger effect in HepG2 (*logFC*=−0.86) than in K562 (*logFC*=−0.13)(Additional file 1: Figure S12).

To examine the biological implications of our results, we followed the analysis performed by Mattioli and colleagues and identified transcription factor binding motifs that are disturbed by the single nucleotide deletions. Focusing only on functional deletions (i.e., deletions that had any effect in one or both cell lines), we looked for DNA binding motifs whose disruptive deletions are over-represented in the set of conditionally functional deletions (i.e., deletions with significantly more effect in one cell line vs. the other) (Fig. 5j). Overall, we found three statistically enriched (Hypgergeometric test, *FDR*<0.05, Methods) motifs in the cell type specific deletions (Fig. 5k, l). Reassuringly, we found that K562-specific deletions were enriched for motifs of the erythroid transcription factor NF-E2. These results demonstrate the potential utility of MPRAnalyze in addressing cases of complex and possibly asymmetric experimental designs.

## Discussion

Massively parallel reporter assays are a powerful technique for functional characterization of enhancer activity in a high-throughput manner. MPRAs can be used to quantify the contribution made by non-coding DNA elements, such as enhancers, to transcriptional activity in nearby genes [10]. It can be further extended to evaluate differences in regulatory activity between different alleles [12], elucidate regulatory grammar via mutagenesis studies [13], and compare enhancer activity between conditions [9]. Complex experimental designs can include interaction studies, where one is interested in how sequence changes affect differential activity between cellular conditions [12], or identifying temporal patterns in time-course data [17].

Since MPRAs are still an actively developing technology, they often vary in experimental design. While MPRAnalyze is flexible and can handle various study designs, the method benefits from certain experimental decisions that are generally recommended but not always leveraged in other analyses. First, pairing the DNA and RNA libraries by extracting DNA from the same post-transduction libraries that the RNA libraries are extracted from, avoids introducing further experimental noise into the data and enables MPRAnalyze to better fit and relate the two models to increase accuracy of estimating nuisance factors. Additionally, as demonstrated in our results, increasing the number of available barcodes and replicates can greatly reduce the measured noise and increase performance of all methods, as seen in recent studies [31, 32]. Finally, the inclusion of negative control sequences allows explicit modeling of the null behavior and avoids relying on assumptions that may bias the results and prevent proper interpretation of them. The curated nature of MPRA datasets makes negative controls a valuable and often crucial aspect of properly interpreting the results.

MPRAnalyze offers a robust statistical framework that enables all major uses of MPRAs in a unified model. Our model avoids relying on ratio-based summary statistics, seeking to directly model the data as structured, following a similar trend in other high throughput functional assay analysis methods, such as recent methods developed for the analysis of deep mutational scanning data [33–35]. MPRAnalyze models noise in both DNA and RNA libraries and uses a nested GLM design to control barcode-specific effects and leverage the multiplicity of barcodes for increased statistical power. The method is highly flexible and allows various complex study designs to be tested in a straight-forward manner, including those currently not supported by any established method. Additionally, MPRAnalyze avoids relying on population-level properties in the analysis, instead leveraging negative controls when available to establish null behaviors.

## Methods

### Dataset collection and processing

For all datasets included in this paper, we relied on the pre-processing and filtering performed by the authors of the original papers. This ensures that MPRAnalyze’s performance isn’t reflecting any favorable processing steps we chose.

For *Kwasnieski*, the study [10] measured the activity of potential regulatory regions in K562 cells. Regions were selected according to ENCODE annotations of four groups: enhancers, weak enhancers, repressed enhancers, enhancers active in ESCs. The repressed and ESC-annotated enhancers were used as controls and were excluded from the analysis after library size normalization factors were computed. In addition to control classes, each class had internal sets of scrambled sequences used as negative controls, which were used as controls in our analyses. Each sequences in this dataset was associated with four barcodes. The DNA was sequenced before transduction and with a single replicate, while four replicates are available for the RNA. While the sample size in this data is very limited, this allowed for higher read counts to be achieved, mitigating the loss of statistical power by getting more reliable quantification of the counts.

For *Inoue-Kircher*, the study [9] compared activity in HepG2 cells of liver enhancers that were either episomal or chromosomally integrated using a lentivirus (lentiMPRA). While the study is comparative, the comparison is not between biological conditions and the results are therefore difficult to validate or interpret. We therefore decided to use the data as two separate quantification datasets. The datasets were analyzed together to better account for batch and barcode-specific effects, and *α* estimates were extracted from the joint model for each condition separately. Negative control sequences were generated by scrambling candidate sequences, and positive controls were sequences that have been previously validated as having a regulatory function in these cells. Each sequence was associated with 100 unique barcodes. DNA was sequenced post-transduction. Both DNA and RNA have three replicates.

For *Inoue-Kreimer*, the study [17] identified enhancers with temporal activity over the first 72 h after neural induction. lentiMPRA was performed in 7 timepoints (0, 3, 6, 12, 24, 48, and 72 h after undiction). For the purpose of our analysis, we used only the data from the first timepoint in the quantification and classification analyses, and timepoints 0 and 72 h for the comparative analysis. Negative controls are scrambled candidate sequences. Each sequence was associated with 90 barcodes. DNA was sequenced post-transduction. Both DNA and RNA have three replicates.

For *Mattioli*, the study [13] compared WT sequences containing core promoters of 31 long non-coding RNAs (21 sequences), enhancer RNAs (5 sequences) and messenger RNAs (5 sequences) with the same sequences with single-nucleotide deletions. Each core promoter was divided to 2 “tiles” to cover more of the putative regulatory sequence, resulting in a total of 62 WT sequences. MPRAs were performed in both K562 and HepG2 cells, with varying number of replicates (4 in K562, 8 in HepG2). DNA was sequenced pre-transduction, and in a single replicate (used for both cell types).

### Computing transcription rate estimates

All transcription rate estimates were computed for library size normalized MPRA data, using upper quartile normalization to compute size factors. *MPRAnalyze’s α* was computed for each dataset using the quantification analysis (See Additional files). Across datasets, batch and barcode-level effects were modelled in the nested DNA model, but excluded from the RNA model design. This allows MPRAnalyze to model nuisance effects but asserts that all barcodes associated with a single sequence must share the same transcription rate. Both ratio-based estimates were computed using only barcodes with non-zero DNA and RNA counts. So for each sequence: *S*={*i*∈[*n*]|*R*_*i*_≠0,*D*_*i*_≠0}. Then the Mean Ratio $=\frac {1}{|S|}\sum _{i\in S}{\frac {R_{i}}{D_{i}}}$, and the Aggregated Ratio $=\frac {\sum _{i\in S}{R_{i}}}{\sum _{j\in S}{D_{j}}}$.

### Running alternative methods

*DESeq2* was used as a method for classifying active enhancers, by comparing the DNA and RNA libraries as the two conditions being compared. DEseq2 was used in two modes: the full mode included each barcode as separate sample, and the collapsed mode took the sum across barcodes within each batch as a sample. In full mode, a single count was added to each observation to avoid issues with DESeq2 library normalization scheme. The model used within DESeq2 was a simple comparison model: type, where type identifies DNA and RNA observations.

*QuASAR-MPRA* was used according to the documentation provided in the package. The *betas.beta.binom* value, which is the logit transformation of the allelic skew, was used as a proxy for log fold-change.

*mpralm* was used according to the documentation provided in the package. For allelic comparisons, while the package vignette uses the sum across barcodes when aggregating the counts, we used the mean across barcodes instead, since the two compared alleles did not have the same number of associated barcodes. Additionally, since the package requires manual aggregation of barcodes in this situation, only the *aggregated* mode of the model is supported for this type of analysis.

### Subsampling analysis

For the subsampling analysis, barcodes were sampled down to varying levels (for Inoue-Kircher datasets: 15, 30, 45, 60, 75, and 90 out of the total 100 barcodes; for Inoue-Kreimer: 15, 30, 45, 60, and 75 of the total 90 barcodes). The analysis uses three independent replicates of this down-sampling process, so overall for each sequence, we get a set of 3×*K* estimates at various numbers of available barcodes, where *K*=6 for the Inoue-Kircher datasets and *K*=5 for Inoue-Kreimer. The analyses were done on the entire down-sampled dataset in a single run and included the original data as well as the reduced-barcodes data, to neutralize any effect that the library size correction might have on the estimates.

### Simulating mPRA data

MPRA data was simulated by generating random coefficients for the nested GLM construction that MPRAnalyze uses. The *latent (true)* DNA and RNA counts were generated directly from the model, then log-normal noise was added to the latent counts to get the *observed* counts. Formally:

$$\begin{array}{*{20}l} \vec{\beta}=&\left[\beta_{0},\vec{\beta_{\text{batch}}},\ \vec{\beta_{\text{BC}}}\right]\\ \beta_{0}\sim&N\left(K, \sigma_{0}^{2}\right)\\ \vec{\beta_{\text{batch}}}\sim&N\left(0, \sigma_{\text{batch}}^{2}\right)\\ \vec{\beta_{BC}}\sim&N\left(0, \sigma_{\text{BC}}^{2}\right)\\ \Downarrow\\ \vec{D}_{\text{true}} =&\ \text{nint}\left(\text{exp}\left(X_{d} \vec{\beta}\right)\right)\\ \vec{R}_{\text{true}} =&\ \text{nint}\left(\text{exp}\left(\alpha\cdot X_{d} \vec{\beta}\right)\right)\\ \vec{D}_{\text{observed}} \sim&\ \text{nint}\left(\mathrm{log-Normal}\left(\text{exp}\left(X_{d} \vec{\beta}\right), \sigma_{D}^{2}\right)\right)\\ \vec{R}_{\text{observed}} \sim&\ \text{nint}\left(\mathrm{log-Normal}\left(\text{exp}\left(\alpha\cdot X_{d} \vec{\beta}\right), \sigma_{R}^{2}\right)\right) \end{array} $$

where *K* controls the intercept term for the construct distribution, the variance of which is $\sigma _{0}^{2}$; $\sigma _{\text {batch}}^{2}, \sigma _{\text {BC}}^{2}$ control the size of batch and barcode effects, respectively; $\sigma _{D}^{2}, \sigma _{R}^{2}$ determine the noise levels added to the data; and nint is the nearest-integer function, using base R’s *round* function. An implementation of this simulation process is included in the MPRAnalyze package.

Noise was generated using log-normal noise instead of Gamma/Negative Binomial to avoid generating data directly from MPRAnalyze’s model, which might bias the results.

Simulated data in this manuscript was generated with three batches, varying numbers of barcodes, *K*=5, and *σ*_0_=*σ*_batch_=*σ*_BC_=*σ*_*D*_=*σ*_*R*_=0.5.

### Transcription factor binding site enrichment analysis

The transcription factor binding site enrichment analysis was performed using the binary binding matrix computed by Inoue and Kreimer et al. [17], with each entry indicating the potential for binding (motif-based binding prediction using Fimo [36], *FDR*<10^−4^) or overlap with transcription factor ChIP-seq peaks from publicly available data [37, 38]. Enrichment was calculated using a hypergeometric test, with all binding motifs of a each transcription factors being pooled together. A factor was deemed enriched if BH-corrected *p* < 0.05. Enrichment scores were calculated as: $\log _{2}\left (\frac {\text {fraction of differential sequences containing a binding site of the TF}}{\text {fraction of total sequences containing a binding st of the TF}}\right).$ For the *consensus + noise* option, for each TF we calculated the number of predicted binding sites in the consensus set and in the remaining population. We then added artificial biding sites to the consensus set, proportional to their abundance in the remaining population, to match the number of differential sequences called by MPRAnalyze.

### Temporal activity analysis

The analysis was performed by setting the full RNA model to include both batch and time-course factors (∼*batch + time*), and the reduced model to batch factors only (∼*batch*). A Likelihood-ratio test is performed for statistical significance, and a sequence is deemed “temporal” if BH-corrected *p*<0.05. Heatmaps for visualization were generated using the ComplexHeatmap R package [39].

### Differential deletions analysis

To identify differential deletions (deletions that affect the induced transcription rate in K562 differently than in HepG2) in the Mattioli dataset we used an interaction term in the RNA model design, encoding the interaction between the cell type factor and the allele factor:

$$\begin{array}{*{20}l} \mathcal{H}_{0}:& \mathrm{RNA \sim Allele + CellType + Allele:CellType}\\ \mathcal{H}_{1}:& \mathrm{RNA \sim Allele + CellType} \end{array} $$

Then a standard likelihood ratio test was performed to determine statistical significance. Since the DNA data has a different design (a single replicate shared across all RNA samples), that design only modeled for barcode specific effects.

### Differential deletion motif analysis

Once differential deletions were identified, we divided the differential deletions to those that had a greater effect in HepG2 or K562. For each cell type, we used the motif hits curated by Mattioli and collegues, which rely on FIMO-based [36] predicted binding scores, to associate each deletion with differential motifs: motifs predicted in one allele and not the other. If the deletion causes a decrease in the induced transcription rate, we took the “lost” motifs (predicted in WT, not in the deletion), and if the deletion caused an increase, we took the “gained” motifs (predicted in the deletion, not the WT). All motifs associated with the same transcription factor were pooled. Enrichment scores were calculated using a hypergeometric test, using the total set of functional deletions as background (motifs for these were acquired in the same fashion).

## Additional files

Additional file 1 Supplemental Figures (S1-12). (PDF 1759 kb)

Additional file 2 Supplemental methods. Formal description of the MPRAnalyze model, hypothesis testing schemes and optimization details. (PDF 231 kb)

Additional file 3 Table s1. Transcription factor binding site enrichment analysis results: BH-corrected *p* values. (CSV 48 kb)

Additional file 4 Table s2. Transcription factor binding site enrichment analysis results: fold-enrichment scores. (CSV 82 kb)

Additional file 5 Table s3. Full RNA-seq measurements from the Inoue-Kreimer study of the timepoints T0h and T72h. (CSV 993 kb)

Additional file 6 Table s4. BH-corrected *p* values from the Transcription Factor finding site enrichment analysis of the temporal results. (CSV 40 kb)

Additional file 7 Review history for the manuscript. (DOCX 50 kb)

## Data Availability

MPRAnalyze is implemented as an R package and is distributed under GPL-3 license through bioconductor [21, 40]: https://bioconductor.org/packages/release/bioc/html/MPRAnalyze.html. Source code and materials used to generate the figures in this paper are available via Zenodo (DOI: 10.5281/zenodo.3345040; https://zenodo.org/record/3345040). Datasets used throughout the manuscript: *Kwasnieski* [10] Data is made available by the authors upon request; *Inoue-Kircher* [9] Available from Gene Expression Omnibus: https://www.ncbi.nlm.nih.gov/geo/query/acc.cgi?acc=GSE83894; *Inoue-Kreimer* [17] Data is made available by the authors upon request; *Mattioli* [13] Available from Gene Expression Omnibus: https://www.ncbi.nlm.nih.gov/geo/query/acc.cgi?acc=GSE117594
