# Supplementary material for: MPRAnalyze: statistical framework for massively parallel reporter assays
Source: Genome Biol. 2019 Sep 2;20:183. doi: 10.1186/s13059-019-1787-z (PMC6717970; doi:10.1186/s13059-019-1787-z)
Supplement: Supplementary file 1 — Supplemental Figures (S1-12). (PDF 1759 kb) [file 13059_2019_1787_MOESM1_ESM.pdf]

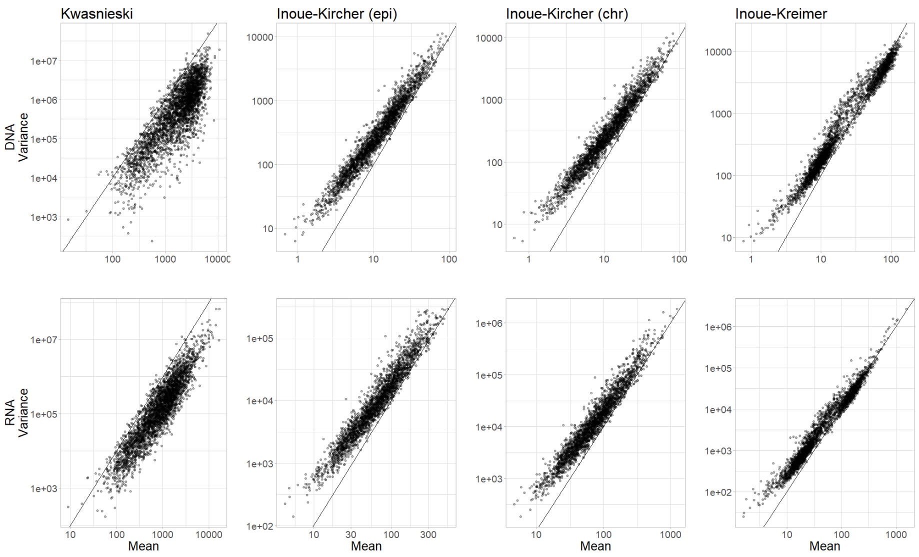

Figure S1: Relationship between the mean and variance of the counts measured for each sequence. Reference line (slope = 2) is a quadratic relationship.

Sample	Condition	Barcode
A	Ref	1
B	Ref	2
C	Ref	3
D	Contrast	1
E	Contrast	2
F	Contrast	3

$$\Rightarrow \log \overbrace{\begin{pmatrix} \widehat{d_A} \\ \widehat{d_B} \\ \widehat{d_C} \\ \widehat{d_D} \\ \widehat{d_E} \\ \widehat{d_F} \end{pmatrix}}^{\text{DNA observations, } \vec{d}} = \overbrace{\begin{bmatrix} 1 & 0 & 0 & 0 \\ 1 & 0 & 1 & 0 \\ 1 & 0 & 0 & 1 \\ 1 & 1 & 0 & 0 \\ 1 & 1 & 1 & 0 \\ 1 & 1 & 0 & 1 \end{bmatrix}}^{\text{Design Matrix, } X_D} \cdot \overbrace{\begin{bmatrix} \beta_0 \\ \beta_{contrast} \\ \beta_{BC_2} \\ \beta_{BC_3} \end{bmatrix}}^{\text{Coefficients, } \vec{\beta}} = \begin{bmatrix} \beta_0 \\ \beta_0 + \beta_{BC_2} \\ \beta_0 + \beta_{BC_3} \\ \beta_0 + \beta_{contrast} \\ \beta_0 + \beta_{contrast} + \beta_{BC_2} \\ \beta_0 + \beta_{contrast} + \beta_{BC_3} \end{bmatrix}$$

$$\Rightarrow \log \overbrace{\begin{pmatrix} \widehat{r_A} \\ \widehat{r_B} \\ \widehat{r_C} \\ \widehat{r_D} \\ \widehat{r_E} \\ \widehat{r_F} \end{pmatrix}}^{\text{RNA observations, } \vec{r}} = \log\left(\vec{d}\right) + \log \overbrace{\begin{pmatrix} \alpha_{ref} \\ \alpha_{ref} \\ \alpha_{ref} \\ \alpha_{constrast} \\ \alpha_{constrast} \\ \alpha_{constrast} \end{pmatrix}}^{\vec{\alpha}} = X_D \cdot \vec{\beta} + \left(\overbrace{\begin{bmatrix} 1 & 0 \\ 1 & 0 \\ 1 & 0 \\ 1 & 1 \\ 1 & 1 \\ 1 & 1 \end{bmatrix}}^{\text{Design Matrix, } X_R} \cdot \overbrace{\begin{bmatrix} \gamma_0 \\ \gamma_{constrast} \end{bmatrix}}^{\text{Coefficients, } \vec{\gamma}} \right) = \begin{bmatrix} \beta_0 \\ \beta_0 + \beta_{BC_2} \\ \beta_0 + \beta_{BC_3} \\ \beta_0 + \beta_{contrast} \\ \beta_0 + \beta_{contrast} + \beta_{BC_2} \\ \beta_0 + \beta_{contrast} + \beta_{BC_3} \end{bmatrix} + \begin{bmatrix} \gamma_0 \\ \gamma_0 \\ \gamma_0 \\ \gamma_0 + \gamma_{constrast} \\ \gamma_0 + \gamma_{constrast} \\ \gamma_0 + \gamma_{constrast} \end{bmatrix}$$

Figure S2: A simplified example of the MPRAnalyze model: two conditions are tested with three barcodes in a paired experiment (each DNA observation has a corresponding RNA observation). No replicates or external normalization factors are included in this design to maintain simplicity. The DNA's model estimation of the latent DNA count, computed as $X_D\vec{\beta}$, is included in the RNA model. The α estimates of transcription rate can be extracted from the model as: $\alpha_{\text{ref}} = e^{\gamma_0}$, $\alpha_{\text{ref}} = e^{\gamma_0 + \gamma_{\text{contrast}}}$. Note that while modeling the barcodes in the RNA model is possible, the result will be a separate α estimator for each barcode, which is usually not desired. Barcode-level information is therefore only incorporated into the RNA model via the nested DNA model.

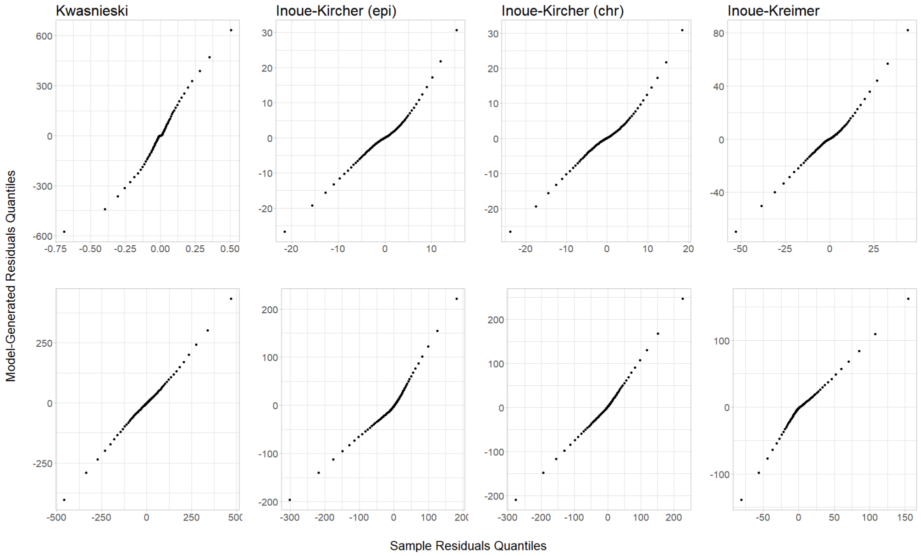

Figure S3: Comparison between model residuals from the observed counts of each dataset, and residuals from random data generated by Gamma (for DNA) and Negative Binomial (for RNA) using the model parameters. Quantile-quantile comparisons indicate that the observed noise and the generated noise follow similar distributions.

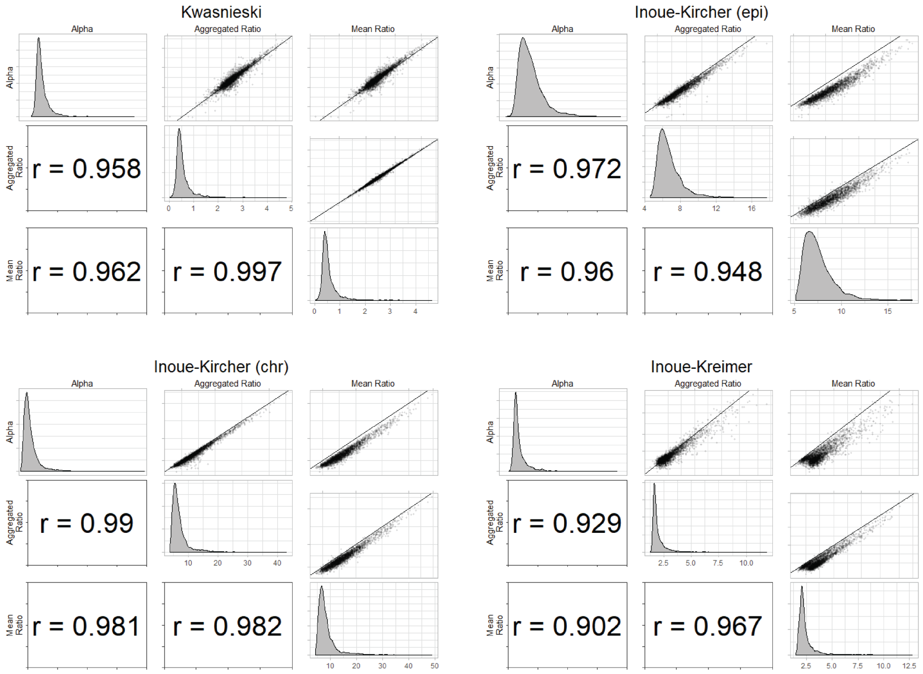

Figure S4: Correlations of MPRAnalyze's *alpha* estimate with the ratio-based estimates. Correlations are Pearson's r.

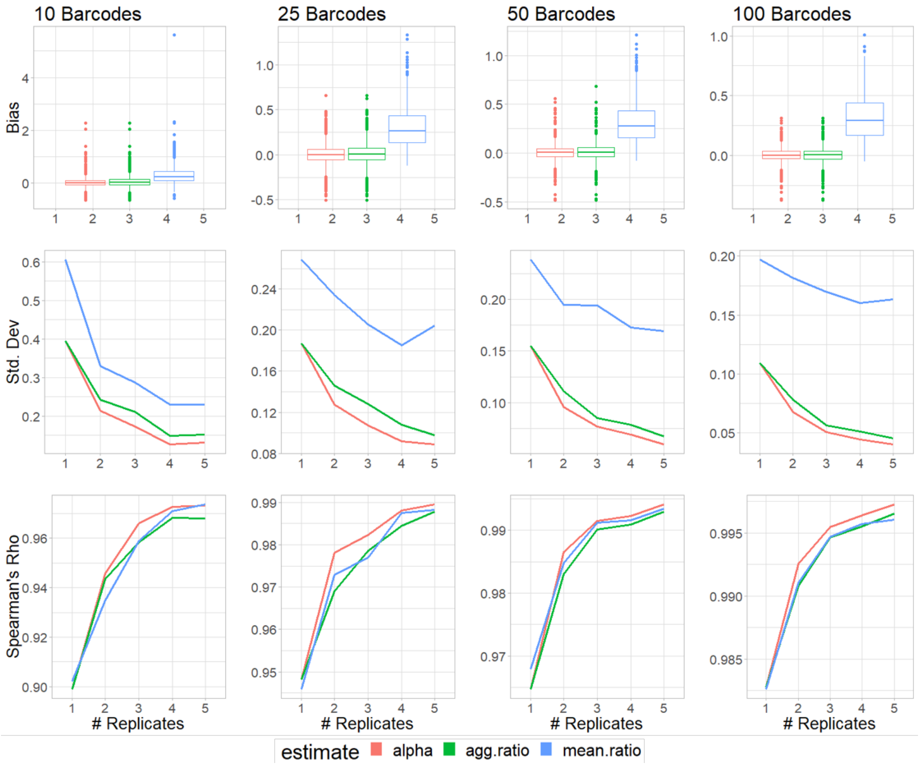

Figure S5: Performance evaluation of MPRAnalyze's α estimate and ratio-based estimates on simulated data with varying number of barcodes and replicates.

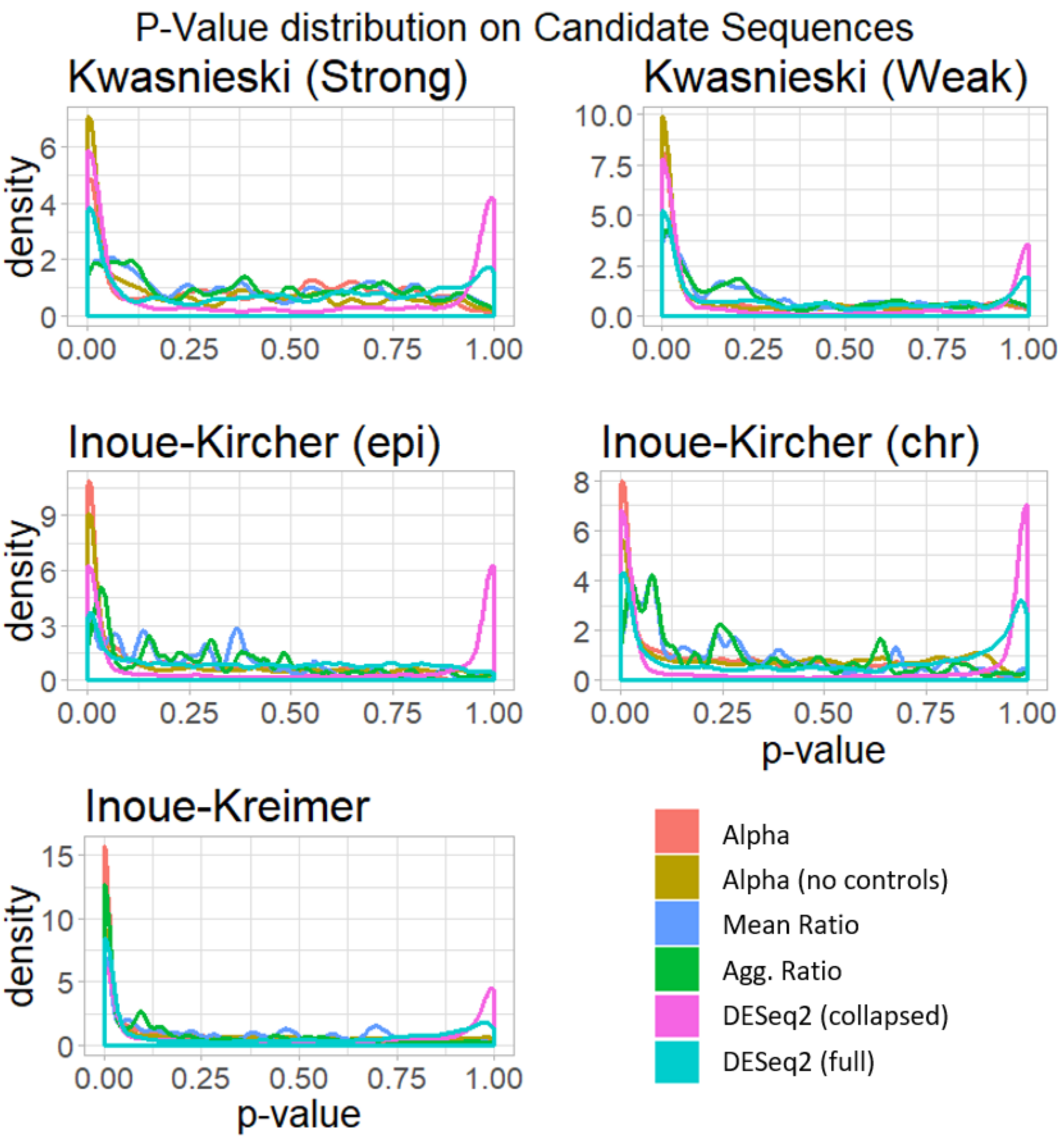

Figure S6: P-value density of classification analysis of candidate sequences for each dataset, by method of classification. Aside from DESeq2-collapsed, all methods seem to follow the theoretical distribution of a mixture of uniformly distributed values and low values.

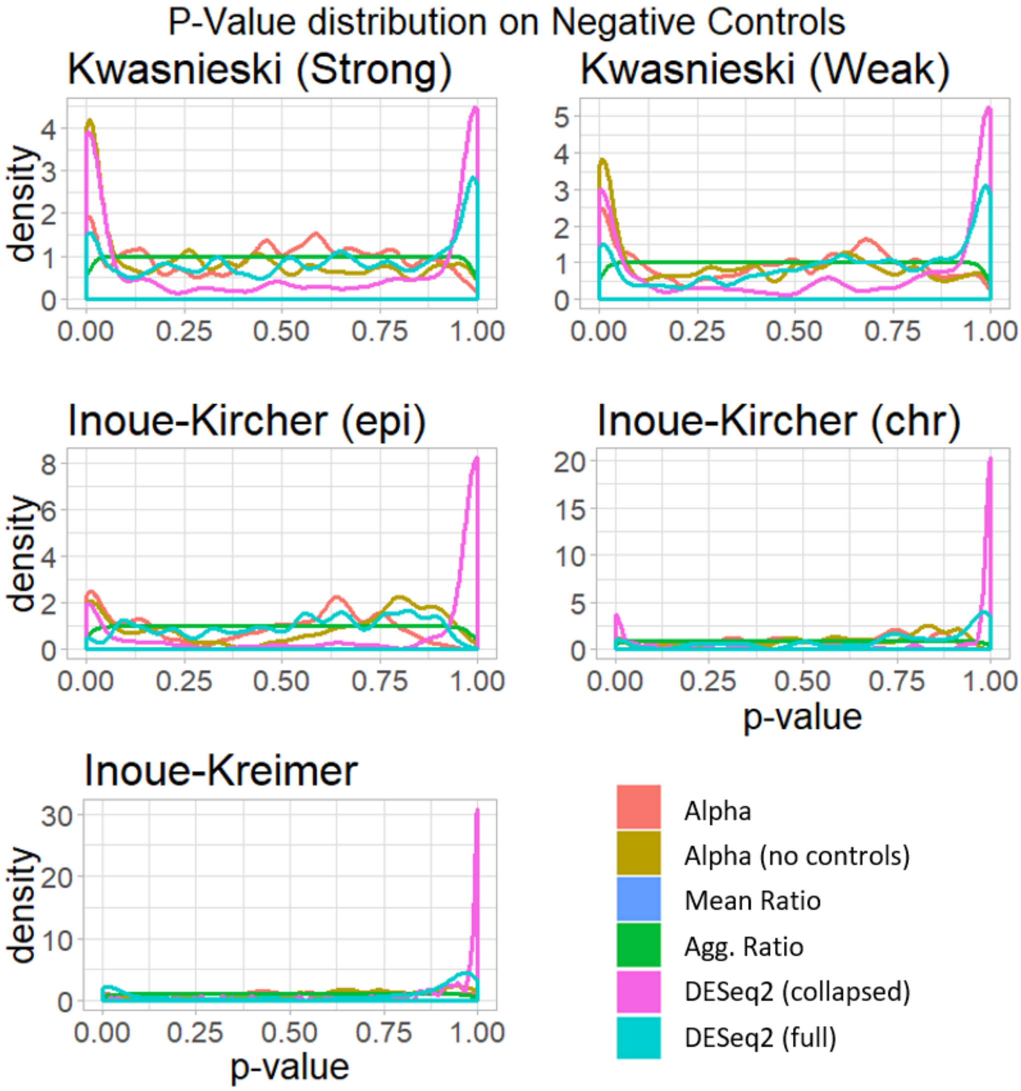

Figure S7: P-value density of classification analysis of negative control sequences for each dataset, by classification method. Empirical methods follow the theoretical uniform y definition, with the Mean Ratio line plotted behind the Agg. Ratio line. Some inflation can be observed with MPRAnalyze in no-controls mode, and DESeq2-collapsed is generally not calibrated.

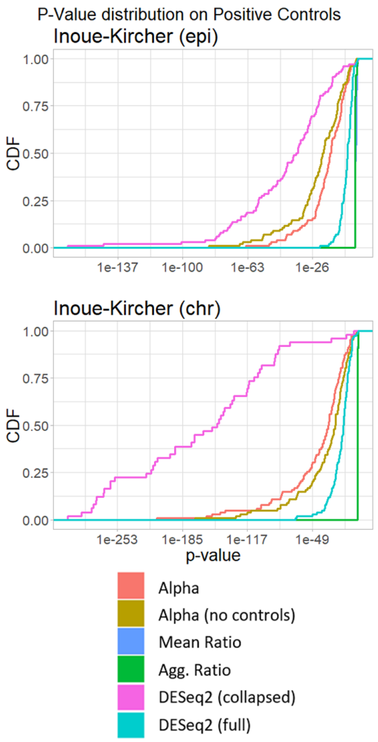

Figure S8: P-value CDF of classification analysis of positive control sequences for each dataset, by classification method, displayed in log scale for ease of visualization. Both modes of MPRAnalyze are substantially more powerful than competing methods, with the exception of DESeq2-collapsed.

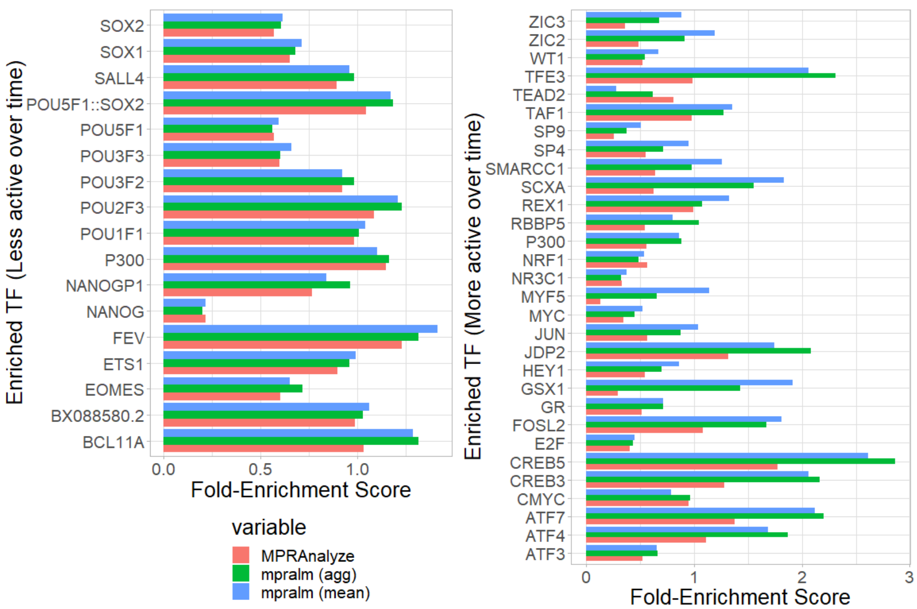

Figure S9: Enrichment score calculated for the set of top-enriched factors (union of top 15 most enriched factors for each method, ranked by statistical score). Enrichment score is calculated as the log2 of the ratio between the fraction of differential sequences that contain a binding motif of that factor, to the fraction of sequences in the entire assay that contain a binding motif for that factor.

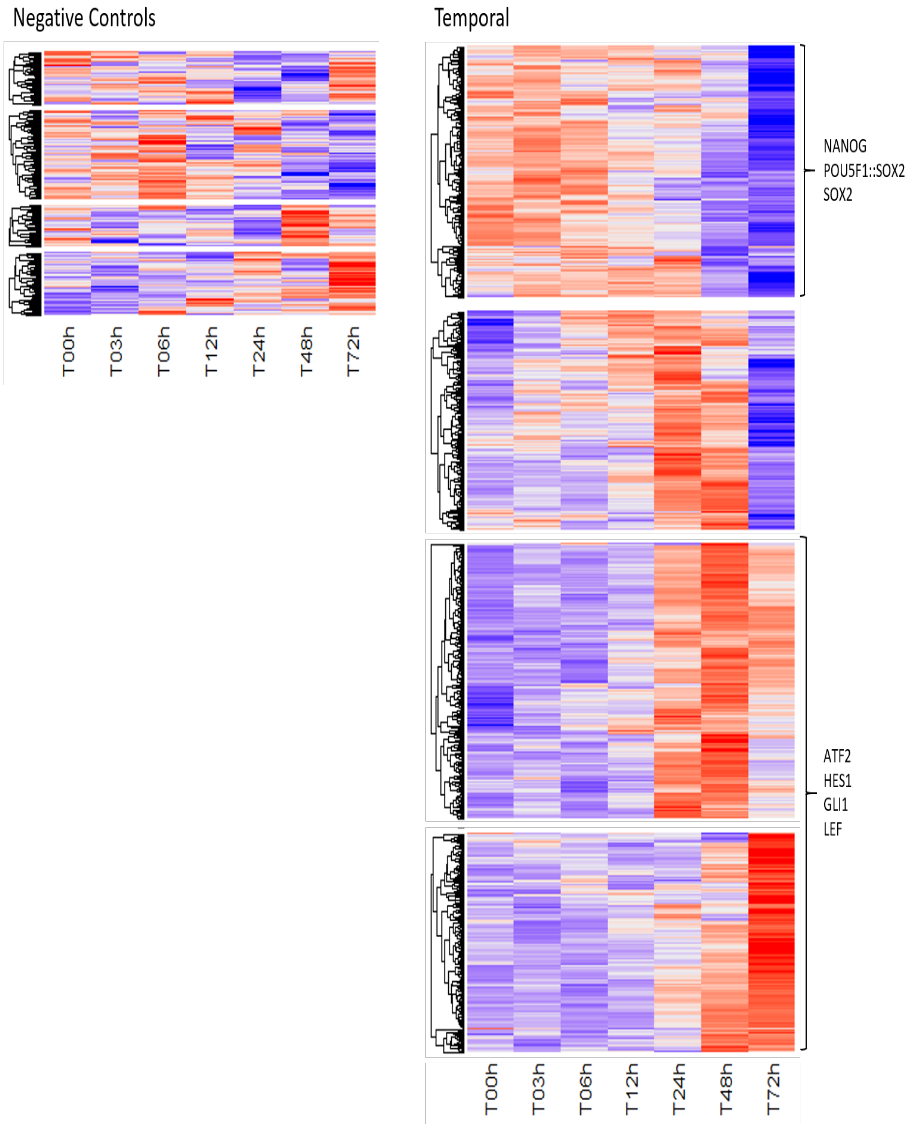

Figure S10: Heatmap of sequence activity (α values) in each time point, for the negative control sequences (left) and sequences that displayed significant temporal activity (right). Rows were z-noramlized for visualization, and each set was clustered using K-Means, K=4, before hierarchical clustering was performed for visualization (gaps indicate distinct clusters). Some enriched transcription factor binding sites are indicated.

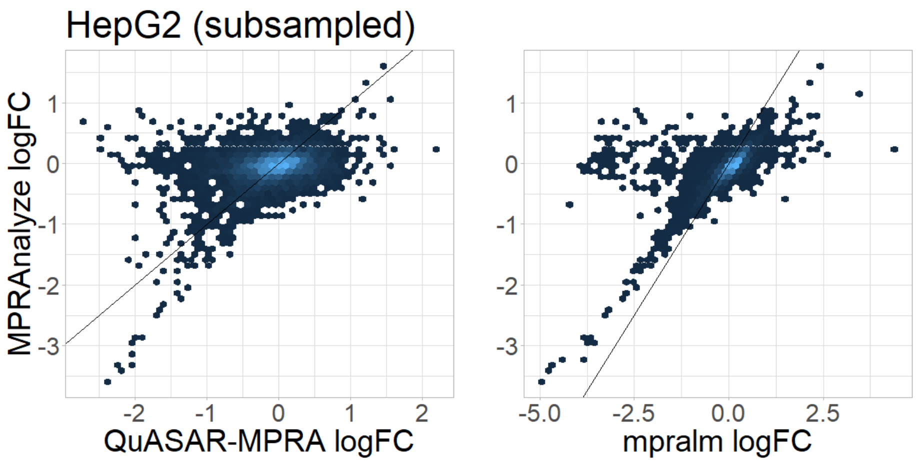

Figure S11: Comparison of log Fold-Change values from competing methods to MPRAnalyze in HepG2 data from the Mattioli study, using only the first 4 replicates.

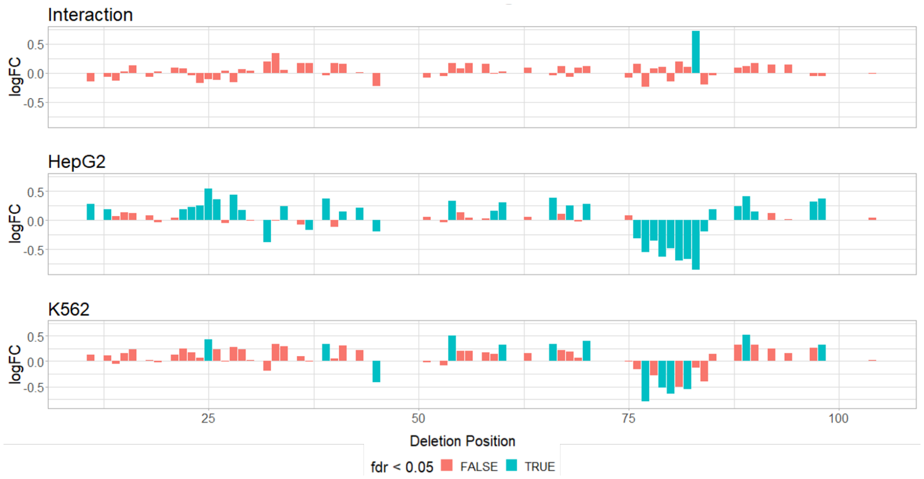

Figure S12: Effect of each single-nucleotide deletion in the core promoter of the lncRNA gene DLEU1. The differential effect size (top) captures the difference in the effect of each deletion in HepG2 (middle) compared with K562 (bottom).
