# Supplementary material for: MPRAnalyze: statistical framework for massively parallel reporter assays
Source: Genome Biol. 2019 Sep 2;20:183. doi: 10.1186/s13059-019-1787-z (PMC6717970; doi:10.1186/s13059-019-1787-z)
Supplement: Supplementary file 7 — Review history for the manuscript. (DOCX 50 kb) [file 13059_2019_1787_MOESM7_ESM.docx]

Review History

**First round of review**

**Reviewer 1**

**Are you able to assess all statistics in the manuscript, including the appropriateness of statistical tests used?**

Comment to author:

Thank you for the opportunity to review this paper. MPRAs and other high-throughput functional assays are an expanding area of biology and good statistical methods are essential for getting the most out of this kind of large dataset.

The authors focus their efforts on the improved statistical properties of MPRAnalyze compared to previous approaches, and these results are quite convincing. However, the paper lacks examples of situations where applying MPRAnalyze would have led to more biologically relevant discoveries.

I have no comments on the code or code availability, since Bioconductor sets a high standard in this area.

Major suggestions

Many of the results are based on analysis of the Inoue-Kreimer dataset [1], but the cited preprint does not have any methods included. It is therefore not possible to compare MPRAnalyze to the method used in the preprint. Because so many of the authors are shared, the authors of this manuscript should clarify the relationship between MPRAnalyze and the missing Inoue-Kreimer methods.

The manuscript lacks sufficient background to place this work in the context of other high-throughput functional assays and would benefit from an expanded introduction and additional references. In particular, the deep mutational scanning (DMS) literature has seen a similar move away from ratios to more advanced methods (e.g. [2] for theory and [3] for theory and implementation). It is possible that this GLM method would have utility for barcoded DMS datasets but that it outside the scope of this paper.

There are a number of unsubstantiated claims that should be backed up (by data or reference) or removed. For example, page 12, lines 12-13 would benefit from an example of a "complex hypothesis" and demonstration that MPRAnalyze's ability to accommodate such hypotheses has practical value. Ideally, this would confirm a finding that is already known in the literature but not tested by the original authors due to limitations in their model. There are numerous similar unsupported statements throughout the manuscript, and the paper would benefit from an additional round of revision by the authors.

Relatedly, throughout the manuscript there are statements that the authors assume are common knowledge and therefore do not need an appropriate citation. This is not only unfair to researchers who deserve to have their contributions cited, but it makes it much harder for students or newcomers to the field to understand the content. For example, on page 12, lines 24-25 the authors state that "In RNA-seq experiments this issue is usually resolved via library size correction" without any reference.

The primary result that demonstrates that the method discovers more "real biology" is based on transcription factor binding site prediction performed by the authors. Are there datasets with independent, low-throughput validation of variants that you can compare to? In its current form, the paper makes a good case that MPRAnalyze is better statistically but fails to show that adopting this method would result in additional biological insights over established methods. 

Minor suggestions

The use of "enhancer" in the paper is a bit confusing, since it refers to individual enhancer variants rather than enhancer targets. This is out of step with other papers in the field, such as [4] in which multiple enhancer targets are assayed. In that preprint, "enhancer" refers to the target and the individual variants are referred to as "mutations."

Does the model make it possible to identify outlier barcodes? That is, barcodes that differ significantly from the population of barcodes tagging a specific variant. Identifying and removing barcodes that may be affecting variant function is a potentially important filtering step for this type of dataset.

Page 5, lines 34-47. This paragraph would benefit from some additional citations justifying the assumptions being made and citing the relevant literature. I agree that "The negative binomial distribution is a common approximation of sequencing data," but the authors should support this statement by citing the relevant literature (e.g. [5,6]).

Why are the ratios called "naive." Is this a technical term? Otherwise it seems like an unjustified slight.

The simulations explore 2-3 fold increased expression. What about decreased expression? The simulations should cover the range that appears in real data.

The section comparing DeSeq2 to MPRAnalyze for classification highlights the importance of having a method built for MPRAs rather than repurposing a DE method. However, it this section would benefit from some restructuring so that the main results are brought forward or the section's results should be summarized at the start. Right now the first result presented, as summarized in Figure 3A, makes the method seem irrelevant compared to DeSeq2.

The broad utility of MPRAnalyze to different MPRA experimental designs could be made more explicit by explaining the experimental designs of datasets analyzed, or simulating according to different designs.

Page 13, line 22 There is an incorrect glyph in the "Pearson's r" statement.

The "Inoue dataset" should be referred to as the "Inoue-Kircher dataset" throughout to acknowledge the contributions of both first authors in line with the Inoue-Kreimer dataset.

Table 1 should include the reference numbers for each study, either as a separate column or as an annotation following the study name.

References

1. Inoue F, Kreimer A, Ashuach T, Ahituv N, Yosef N. Massively parallel characterization of regulatory dynamics during neural induction. bioRxiv. 2018;370452. 
2. Matuszewski S, Hildebrandt ME, Ghenu A-H, Jensen JD, Bank C. A Statistical Guide to the Design of Deep Mutational Scanning Experiments. Genetics. 2016;204:77-87. 
3. Rubin AF, Gelman H, Lucas N, Bajjalieh SM, Papenfuss AT, Speed TP, et al. A statistical framework for analyzing deep mutational scanning data. Genome Biol. 2017;18:150. 
4. Kircher M, Xiong C, Martin B, Schubach M, Inoue F, Bell RJ, et al. Saturation mutagenesis of disease-associated regulatory elements. bioRxiv. 2018;505362. 
5. Lönnstedt I, Speed T. REPLICATED MICROARRAY DATA. Stat Sin. 2002;12:31-46. 
6. Robinson MD, McCarthy DJ, Smyth GK. edgeR: a Bioconductor package for differential expression analysis of digital gene expression data. Bioinformatics. 2010;26:139-40.

**Reviewer 2**

**Are you able to assess all statistics in the manuscript, including the appropriateness of statistical tests used?** Yes, to my knowledge, their statistical framework and choice of methods is adequate.

Comment to author:

The authors of this work describe a new statistical framework to analyze MPRA data. The publication of a flexible method to analyze MPRA data is timely. Current available methods were either limited in the kind of analysis that they can perform (i.e QuASAR) or they were not set for MPRA data analysis and thus if used on MPRA data some assumptions were violated (i.e DESeq2). MPRAnalyze uses a flexible model that allows testing different questions, such as comparing activities within sequences of the same experiment, or across experiments. MPRAnalize also takes advantage of the use of many barcodes to increase its power to detect activity or to perform differential activities analysis. The authors compare MPRAnalyze with available methods thoroughly and they highlight the advantages of one method versus the others. Overall, the methods used are appropriate to the aims of the study and they are well justified across the manuscript. I believe this method will be very useful to the scientific community. 

We have run MPRAnalyze to analyze our own datasets. It has been easy to download, install and run. However, I have some concerns regarding the applicability of the method in different contexts (see comments below), and general comments about the manuscript that should be addressed before publication. 

Major comments:

1- The performance of MPRAnalyze for analyzing allelic data should be tested. Many applications of MPRA are focused on finding regulatory variants and thus the authors should add a section where allelic datasets are analyzed. Also, methods such as QuASAR where designed to analyze SNPs and thus, it should be evaluated in this context. There are several publications with this kind of data available such as Tewhey et al. 2016 Cell, Ulirsch et al. 2016 Cell, Mattioli et al. GenRes 2019, among others. Also, there is no vignette explaining how to analyze this kind of datasets. 

2- Can MPRAnalyze be run on repressive sequences? It is unclear to me if it would be able to find sequences that have a close to zero alpha estimate even if the MPRA were run with strong promoters and the corresponding negative control distributions. 

3- The authors comment on the gain of power by adding more barcodes but they do not comment or analyze this in the context of the number of replicates. A similar analysis done with the barcodes but with the number of replicates would be useful. 

4- Expression of TFs should be assessed across all TF enriched (analysis performed in page 12), and not only for the two TFs that MPRAnalyze finds enriched compared to mpralm.

5- additional information table 1 should be added such as 1) the # of Negative controls (and maybe also how those negative controls were generated, are they random? scrambled?), 2) the # of replicates and 3) whether the DNA was sequenced pre- or post-transduction

6- Add a methods section for DESeq2. Specific question: when running DESeq2 in "full" mode (i.e. per barcode), how do they arrive at a p-value per enhancer?

7- Re: Figure 1: Is Kwasnieski dataset modeled the best because it's sequenced pre-transduction? Why are the differences with the data in this study compared to the others so large (Fig 1A)?

8- Page 8, line 36. With regard to the statement: Both DESeq2-collapsed and mPRAnalyze without controls have inflated rates of activity in the Kwasnieski datasets. What threshold (fraction of active sequences) do you use to make this statement? It is unclear to me how do we know what the true fraction is. Please clarify.

9- the labels in figure 3A for DESeq collapsed and aggregate mean are swapped. Or else the statement that "MPARanlyze and DESeq2 find comparable number of enhaners" does not make sense. Please fix/clarify. What does MPRAnalyze-NC means? Negative controls or No controls? It would be best NOT to abbreviate any of the names in the legend.

10- Figure S5 should be divided in different panels and appropriately cited in the text, page 8, starting in line 42. For example, the statement " MPRAAnalyse model is far better calibrated to MPRA data", does this to Fig S5 middle panel? Then specify. Also, I see that the "mean ratio" fits very well the theoretical line in the middle panel, please explain. Finally, figure S5, middle and bottom, has "DESeq2 collapsed" line missing, I do not see it, maybe is behind other lines but should be clarified in the legend. Histograms (or density plots) would be more informative than CDFs, since it is the way p-value distributions are generally represented. 

11- Figure S6 is confusing and it is not clear to me what it represents. Please explain either in the legend or in the text 


Minor comments:

- Figure 3C specify that the "controls" are negative in the figure by adding "negative controls". 

- Typos
1. Page 2, line 54: "it's" should be "its"
2. Page 7, line 40: "FIgure" should be "Figure"
3. Page 11, line 22: upside down question mark should be >
4. Page 27, line 20: "nhancers" should be "enhancers"

- Some supplementary figures have extreme small text labels, please fix. Most main figures have different font sizes across plots. 

Marta Melé
Group Leader
Transcriptomics and Functional Genomics Lab
Life Sciences Department
Barcelona Supercomputing Center

**Reviewer 3**

**Are you able to assess all statistics in the manuscript, including the appropriateness of statistical tests used?** Yes: Methods are clear and well written.

Comments to author:

The manuscript "MPRAnalyze - A statistical framework for Massively Parallel Reporter Assays" by Ashuach et al. presents statistical model for the analysis of reporter assay data and its implementation as R bioconductor package. The method, underlying assumptions and the relation to available tools are very well described in the manuscript. Overall, the manuscript is timely and of interest to the community. My comments are mostly in regards to the structure and some specific details of the manuscript.

- The first two paragraphs of the introduction are lengthy and have too little focus on the actual topic. A more relevant introduction seems to be hiding in the first paragraph of the results section.

- I would recommend replacing the term enhancer with "regulatory sequence"/"sequence" throughout the manuscript. The definition of the term enhancer is somewhat unclear and introduces an unnecessary and artificial limitation of MPRAs and this method.

- p5, l28-29: barcode-level effects on RNA; I do not agree with this statement. Barcodes could easily influence UTR stability and RNA decay, thereby affecting transcriptional activity. Further, (and similar to the DNA) the barcode will also effect amplification efficiencies in library prep.

- p7/Fig.2 & 3: I am wondering whether labeling and description of agg.ratio/mean ratio is consistent throughout the figures and the text. It was unexpected for me that, the mean ratio method is supposed to have the lower variance, while being described earlier as most sensitive to noise. I would ask that a short description of agg. ratio/mean ratio is included in the figure legends, for example the formulas given in the methods.

- p11, l15: correct citation
- p 11,l16: introduce BH/Benjamini-Hochberg abbreviation at first mention
- p 11,l22: fix incorrect character after "Pearson's r"

-p 11,l46ff/Supp. Fig 7: analysis seems over the top for this purpose. Consider removing/moving to the supplement.

- legend Figure 1: "plots for both libraries" -> "plots for DNA and RNA libraries"

**Point-by-point responses to the reviewers’ comments:**

**Referee 1**

Thank you for the opportunity to review this paper. MPRAs and other high-throughput functional assays are an expanding area of biology and good statistical methods are essential for getting the most out of this kind of large dataset.

The authors focus their efforts on the improved statistical properties of MPRAnalyze compared to previous approaches, and these results are quite convincing. However, the paper lacks examples of situations where applying MPRAnalyze would have led to more biologically relevant discoveries.

I have no comments on the code or code availability, since Bioconductor sets a high standard in this area.

Major suggestions

Many of the results are based on analysis of the Inoue-Kreimer dataset [1], but the cited preprint does not have any methods included. It is therefore not possible to compare MPRAnalyze to the method used in the preprint. Because so many of the authors are shared, the authors of this manuscript should clarify the relationship between MPRAnalyze and the missing Inoue-Kreimer methods.

***Response****: We thank the reviewer for the comment. The original analysis for the Inoue-Kreimer study used an early version of the model that later evolved into the MPRAnalyze model and package. That preliminary model had a similar structure of nested generalized linear models, but used different distributional assumptions (log-normal for both DNA and RNA libraries, instead of the gamma-poisson model used by MPRAnalyze). This option is still supported in the package, and is thoroughly described in the MPRAnalyze supplemental methods, under ‘Distributional Models (2.2)’. We have now repeated the analysis of the Inoue-Kreimer dataset, using all seven time points, in order to demonstrate the utility of MPRAnalyze in this type of study design (see pages 12-13 and new figure S10).*

The manuscript lacks sufficient background to place this work in the context of other high-throughput functional assays and would benefit from an expanded introduction and additional references. In particular, the deep mutational scanning (DMS) literature has seen a similar move away from ratios to more advanced methods (e.g. [2] for theory and [3] for theory and implementation). It is possible that this GLM method would have utility for barcoded DMS datasets but that it outside the scope of this paper.

***Response****: We thank the review for pointing out this important body of literature.  We now mention this and cite these papers in the discussion section (Page 16, line 30)*

There are a number of unsubstantiated claims that should be backed up (by data or reference) or removed. For example, page 12, lines 12-13 would benefit from an example of a "complex hypothesis" and demonstration that MPRAnalyze's ability to accommodate such hypotheses has practical value. Ideally, this would confirm a finding that is already known in the literature but not tested by the original authors due to limitations in their model. There are numerous similar unsupported statements throughout the manuscript, and the paper would benefit from an additional round of revision by the authors.

***Response****: An important feature of MPRAnalyze is that it provides a flexible statistical framework that can model the effects of various experimental covariates on the MPRA output. Following the reviewere’s suggestion, we have included two additional analyzes in the manuscript. (1) We use MPRAnalyze to identify temporally-activated sequences in the Inoue-Kreimer data. This analysis requires modeling a multi-covariate condition (time-course), and cannot be performed by any of the competing methods. We found that temporal sequences that show decreased activation over time are enriched for binding motifs of pluripotent factors (NANOG, SOX2, POU5F1), and that sequences that show increased activation over time are enriched for neural transcription factors (ATF1, HES1, GLI1, LEF). (2) Using data from Mattioli and collegues, we tested for interaction between cell type and allele (wild-type against single nucleotide deletion). Our results, as demonstrated in the manuscript, found that deletions that had a K562-specific effect (more effect in K562 than in HepG2) were enriched for the loss or gain of the binding motif of NF-E2, an erythroid transcription factor.*

*Both examples demonstrate MPRAnalyze’s utility in complex study analysis and the ability to re-discover known and established biology. To further address the reviewer’s concerns, we have extensively edited the manuscript, to make it overall clearer and more concise.*

Relatedly, throughout the manuscript there are statements that the authors assume are common knowledge and therefore do not need an appropriate citation. This is not only unfair to researchers who deserve to have their contributions cited, but it makes it much harder for students or newcomers to the field to understand the content. For example, on page 12, lines 24-25 the authors state that "In RNA-seq experiments this issue is usually resolved via library size correction" without any reference.

***Response****: We have now added the missing citation and scanned the paper for similar cases in page 2, line 7 and page 2, line 40. In this revision, we haver aimed to account for similar cases throughout the text.*

The primary result that demonstrates that the method discovers more "real biology" is based on transcription factor binding site prediction performed by the authors. Are there datasets with independent, low-throughput validation of variants that you can compare to? In its current form, the paper makes a good case that MPRAnalyze is better statistically but fails to show that adopting this method would result in additional biological insights over established methods.

***Response****:*

1. *We thank the reviewer for the suggestion of looking at independent, low-throughput validations as a way of assessing the algorithm. We are currently employing this approach at the level of an entire region by inspecting the output of MPRAnalyze (and other methods) on positive controls (namely, regions that are validated enhancers in the respective model system). We find that MPRAnalyze is capable of identifying those regions effectively, compared to other methods (see Figures 3A, S8). We have now supplemented this analysis by using MPRAnalyze to identify temporal behavior in the full Inoue-Kreimer dataset (Figure S9) and to look for interactions between cellular context and genetic perturbations (Figure S11), an analysis that is not supported by any of the existing methods.*
2. *The TFBS analysis is based both of prediction (using FIMO) and validated hits (using collected ChIP-seq data) curated in the Inoue-Kreimer study. We demonstrate that MPRAnalyze’s increased statistical power enables discovery of two transcription factors that have been implicated in neural development, but  are not identified by any of the established methods (Figures 4D, S9).*

Minor suggestions

The use of "enhancer" in the paper is a bit confusing, since it refers to individual enhancer variants rather than enhancer targets. This is out of step with other papers in the field, such as [4] in which multiple enhancer targets are assayed. In that preprint, "enhancer" refers to the target and the individual variants are referred to as "mutations."

***Response****: We appreciate and agree with the reviewer’s comments, and replaced references to sequences tested in an MPRA experiment from ‘enhancer’ to ‘sequence’ where appropriate.*

Does the model make it possible to identify outlier barcodes? That is, barcodes that differ significantly from the population of barcodes tagging a specific variant. Identifying and removing barcodes that may be affecting variant function is a potentially important filtering step for this type of dataset.

***Response****: We thank the reviewer for the comment. Identifying outlier barcodes is a preprocessing step that is left up to the user, since the definition of an “outlier barcode” can change between experiments. If the barcode maintains the correct ratio (e.g if it has an unusually high\low count in both dna and rna libraries), then modeling barcode effects in the MPRAnalyze framework will account for this. If the barcode is an outlier in terms of the ratio, then with enough additional barcodes it should have minimal effect on the alpha estimation. If the number of additional barcodes is limited, then the definition of an outlier barcode becomes increasingly problematic.*

Page 5, lines 34-47. This paragraph would benefit from some additional citations justifying the assumptions being made and citing the relevant literature. I agree that "The negative binomial distribution is a common approximation of sequencing data," but the authors should support this statement by citing the relevant literature (e.g. [5,6]).

***Response****: We thank the reviewer for the comment, and have added an appropriate citation for this claim. Other claims in this paragraph refer to basic mathematical properties of the Negative Binomial and Gamma distributions, and are therefore stated without citation.*

Why are the ratios called "naive." Is this a technical term? Otherwise it seems like an unjustified slight.

***Response****: We appreciate the reviewer’s comment. By “Naive” we intended to refer to the most obvious and intuitive solution that is not necessarily optimal. We have now removed any use of the term ‘naive’ from the manuscript.*

The simulations explore 2-3 fold increased expression. What about decreased expression? The simulations should cover the range that appears in real data.

***Response****: We have now reran the simulation analysis with transcription rates ranging from 0.2 to 3 (in 0.01 steps), spanning both increased (>>1) and decreased (<<1) expression of RNA relative to DNA. Figures 2E-G were regenerated with the new results, with no substantial change to the interpretation. We did not observe any substantial changes in the results when examining decreased expression compared with increased expression.*

The section comparing DeSeq2 to MPRAnalyze for classification highlights the importance of having a method built for MPRAs rather than repurposing a DE method. However, it this section would benefit from some restructuring so that the main results are brought forward or the section's results should be summarized at the start. Right now the first result presented, as summarized in Figure 3A, makes the method seem irrelevant compared to DeSeq2.

***Response****: We thank the reviewer for this suggestion and have edited the opening paragraph on the classification section to explicitly mention these results, as suggested.*

The broad utility of MPRAnalyze to different MPRA experimental designs could be made more explicit by explaining the experimental designs of datasets analyzed, or simulating according to different designs

***Response****: We have now included details of the different designs in Table 1 and further provide a more detailed description in the Methods section. We note that the dataset and analysis we added in this revision (from Mattioli et al.) has several levels of asymmetry in the design, which are also described and addressed in the manuscript. Out added analysis of temporal behavior using the Inoue-Kreimer data provides further examples of different designs.*

Page 13, line 22 There is an incorrect glyph in the "Pearson's r" statement.

***Response****: We have corrected this.*

The "Inoue dataset" should be referred to as the "Inoue-Kircher dataset" throughout to acknowledge the contributions of both first authors in line with the Inoue-Kreimer dataset.

***Response****: We agree with the reviewer and have corrected this.*

Table 1 should include the reference numbers for each study, either as a separate column or as an annotation following the study name.

***Response****: The citations in Table 1 were moved from the description column to the study name column, to make finding the corresponding studies easier.*

References

1. Inoue F, Kreimer A, Ashuach T, Ahituv N, Yosef N. Massively parallel characterization of regulatory dynamics during neural induction. bioRxiv. 2018;370452.

2. Matuszewski S, Hildebrandt ME, Ghenu A-H, Jensen JD, Bank C. A Statistical Guide to the Design of Deep Mutational Scanning Experiments. Genetics. 2016;204:77-87.

3. Rubin AF, Gelman H, Lucas N, Bajjalieh SM, Papenfuss AT, Speed TP, et al. A statistical framework for analyzing deep mutational scanning data. Genome Biol. 2017;18:150.

4. Kircher M, Xiong C, Martin B, Schubach M, Inoue F, Bell RJ, et al. Saturation mutagenesis of disease-associated regulatory elements. bioRxiv. 2018;505362.

5. Lönnstedt I, Speed T. REPLICATED MICROARRAY DATA. Stat Sin. 2002;12:31-46.

6. Robinson MD, McCarthy DJ, Smyth GK. edgeR: a Bioconductor package for differential expression analysis of digital gene expression data. Bioinformatics. 2010;26:139-40.

**Reviewer 2**

The authors of this work describe a new statistical framework to analyze MPRA data. The publication of a flexible method to analyze MPRA data is timely. Current available methods were either limited in the kind of analysis that they can perform (i.e QuASAR) or they were not set for MPRA data analysis and thus if used on MPRA data some assumptions were violated (i.e DESeq2). MPRAnalyze uses a flexible model that allows testing different questions, such as comparing activities within sequences of the same experiment, or across experiments. MPRAnalize also takes advantage of the use of many barcodes to increase its power to detect activity or to perform differential activities analysis. The authors compare MPRAnalyze with available methods thoroughly and they highlight the advantages of one method versus the others. Overall, the methods used are appropriate to the aims of the study and they are well justified across the manuscript. I believe this method will be very useful to the scientific community.

We have run MPRAnalyze to analyze our own datasets. It has been easy to download, install and run. However, I have some concerns regarding the applicability of the method in different contexts (see comments below), and general comments about the manuscript that should be addressed before publication.

Major comments:

1- The performance of MPRAnalyze for analyzing allelic data should be tested. Many applications of MPRA are focused on finding regulatory variants and thus the authors should add a section where allelic datasets are analyzed. Also, methods such as QuASAR where designed to analyze SNPs and thus, it should be evaluated in this context. There are several publications with this kind of data available such as Tewhey et al. 2016 Cell, Ulirsch et al. 2016 Cell, Mattioli et al. GenRes 2019, among others. Also, there is no vignette explaining how to analyze this kind of datasets.

***Response****: We thank the reviewer for this comment and suggestions and agree that this is a valuable application of MPRAs that is important to address in our manuscript. We therefore: (1) added a subsection that deals with allelic comparison using the data from Mattioli et al. We find similar trends as the comparative settings: QuASAR-MPRA is not well calibrated, and MPRAnalyze shows more statistical power than alternative methods. (2) the most recent version of the package Vignette now also contains a brief explanation on running allelic comparisons with MPRAnalyze.*

2- Can MPRAnalyze be run on repressive sequences? It is unclear to me if it would be able to find sequences that have a close to zero alpha estimate even if the MPRA were run with strong promoters and the corresponding negative control distributions.

***Response****: To address this, we have now reran the simulation analysis with transcription rates ranging from 0.2 to 3 (in 0.01 steps), spanning both increased (>>1) and decreased (<<1) expression of RNA relative to DNA. Figures 2E-G were regenerated with the new results, with no substantial change to the interpretation. We found that while absolute bias and variance values are smaller with low transcription rates, the comparison between methods and the overall trends are virtually identical.*

*Notably, this classification analysis relies on our quantification model, which can estimate any transcription rate as long as sufficient data is available (though zero counts are generally impossible to distinguish from technical problems). In the case described by the reviewer, the alpha value would be significantly lower than the control distribution, and will be classified as significant by the two-sided test.*

3- The authors comment on the gain of power by adding more barcodes but they do not comment or analyze this in the context of the number of replicates. A similar analysis done with the barcodes but with the number of replicates would be useful.

***Response****: We thank the reviewer for this comment and agree that this analysis could be useful in informing experimental design of future MPRAs. To address the effect of replicate number, we have now simulated data with varying number of barcodes and replicates, and visualized the results in Supplementary Figure 5. We find that the number of replicates, like the number of barcodes, increases performance of all estimates, with similar trends: MPRAnalyze’s alpha performing better than the aggregate ratio, and both outperforming the mean ratio. We also find that increasing the number of replicates can mitigate a limited number of barcodes, for instance: an experiment with 10 barcodes per sequence and 3 replicates has similar performance, across estimates, as an experiment with 25 barcodes in a single replicate.*

4- Expression of TFs should be assessed across all TF enriched (analysis performed in page 12), and not only for the two TFs that MPRAnalyze finds enriched compared to mpralm.

***Response****: We thank the reviewer for this comment. We ran a Differential Expression Analysis on the RNA-seq data to generate results for the entire set of enriched factors. While we do observe the expected signal in the main pluripotency and neural transcription factors, we found that the results in general are not consistent with our enrichment analysis. This can be due to similarities between motifs of different TFs or due to discrepancy between mRNA levels and protein- level activity of TFs. We therefore removed the differential expression analysis from the manuscript.*

5- additional information table 1 should be added such as 1) the # of Negative controls (and maybe also how those negative controls were generated, are they random? scrambled?), 2) the # of replicates and 3) whether the DNA was sequenced pre- or post-transduction

***Response****: We thank the reviewer for this comment, and all suggested information has been added to the table. Since all negative controls in all datasets were scrambled, this was excluded from the table and included in the more detailed methods section for each study. Additionally, for formatting reasons, the description of the datasets was moved to the methods section.*

6- Add a methods section for DESeq2. Specific question: when running DESeq2 in "full" mode (i.e. per barcode), how do they arrive at a p-value per enhancer?

***Response****: DESeq2 in ‘full’ mode used each barcode as a separate sample, but the anaysis was still conducted at the enhancer level (whereas the collapsed mode took the sum across barcode within each batch). We have now clarified this in the main text (p. 8) and methods section (p. 39)*

7- Re: Figure 1: Is Kwasnieski dataset modeled the best because it's sequenced pre-transduction? Why are the differences with the data in this study compared to the others so large (Fig 1A)?

***Response****: We thank the reviewer for the comment. The Kwasnieski data is limited by only having four barcodes associated with each sequence, and only having a single replicate in the DNA library. Therefore the DNA model, when taking barcode-specific effects into account, can reach a perfect fit. In this case the estimates of the DNA counts, used in the RNA model, are identical to the observed DNA counts. The small sample size in terms of barcodes allowed for much higher read counts per barcode, which explains the major differences in Figure 1A. These clarifications have been included in the text (p. 6).*

8- Page 8, line 36. With regard to the statement: Both DESeq2-collapsed and MPRAnalyze without controls have inflated rates of activity in the Kwasnieski datasets. What threshold (fraction of active sequences) do you use to make this statement? It is unclear to me how do we know what the true fraction is. Please clarify.

***Response****: We thank the reviewer for the comment. That statement is based on the fraction of negative controls found to be activity, compared with an expected 5% false discovery rate. This has been clarified in the text as well (p. 9, line 10).*

9- the labels in figure 3A for DESeq collapsed and aggregate mean are swapped. Or else the statement that "MPARanlyze and DESeq2 find comparable number of enhaners" does not make sense. Please fix/clarify. What does MPRAnalyze-NC means? Negative controls or No controls? It would be best NOT to abbreviate any of the names in the legend.

***Response****: We thank the reviewer for this correction. The legend in Figure 3A indeed mislabeled some of the results. The legend was fixed and abbreviations were replaced with explicit labels (NC = no controls).*

10- Figure S5 should be divided in different panels and appropriately cited in the text, page 8, starting in line 42. For example, the statement " MPRAAnalyse model is far better calibrated to MPRA data", does this to Fig S5 middle panel? Then specify. Also, I see that the "mean ratio" fits very well the theoretical line in the middle panel, please explain. Finally, figure S5, middle and bottom, has "DESeq2 collapsed" line missing, I do not see it, maybe is behind other lines but should be clarified in the legend. Histograms (or density plots) would be more informative than CDFs, since it is the way p-value distributions are generally represented.

***Response****: We appreciate the reviewer’s comment and have reworded and revisualized this section accordingly. The supplementary plot was split into three panels (candidate sequences, negative controls and positive controls, Figures S6, S7 and S8 respectively) that are referred to in the text separately and explicitly (p. 9). The legend was incorrect, switching the DESeq2-collapsed color with the mean ratio color (which explains both odd results the reviewer mentioned: DESeq2 is very powerful when measuring positive controls, but is not well calibrated generally. The missing line is the mean-ratio, which in both control cases has the exact same performance as the aggregated ratio, and is plotted under it. This has been clarified in the figure legends). The plots for the candidate and negative controls were replaced with density plots, whereas the positive controls remain CDFs and in log scale to make them more informative.*

11- Figure S6 is confusing and it is not clear to me what it represents. Please explain either in the legend or in the text

***Response****: We have now edited the figure, caption and text to better deliver the message underlying this figure. The figure is meant to demonstrate that DESeq2 is not well calibrated despite an overall agreement in statistical scores with MPRAnalyze (meaning the abundant high p-values in DESeq2 results are associated with non-significant p-values in the MPRAnalyze results as well, which is why the overall numbers of significant sequences is still similar), due to the assumptions that the DESeq2 model makes that don’t hold for MPRA data (namely, that the differential signal is symmetric).*

Minor comments:

- Figure 3C specify that the "controls" are negative in the figure by adding "negative controls".

***Response****: We agree and have corrected the figure.*

- Typos

1.	Page 2, line 54: "it's" should be "its"

2.	Page 7, line 40: "FIgure" should be "Figure"

3.	Page 11, line 22: upside down question mark should be >

4.	Page 27, line 20: "nhancers" should be "enhancers"

***Response****: We thank the reviewer for these corrections, and have applied all to the text.*

- Some supplementary figures have extreme small text labels, please fix. Most main figures have different font sizes across plots.

***Response****: We thank the reviewer for pointing this out, and have adjusted font size in all figures to be larger and consistent.*

**Reviewer 3**

The manuscript "MPRAnalyze - A statistical framework for Massively Parallel Reporter Assays" by Ashuach et al. presents statistical model for the analysis of reporter assay data and its implementation as R bioconductor package. The method, underlying assumptions and the relation to available tools are very well described in the manuscript. Overall, the manuscript is timely and of interest to the community. My comments are mostly in regards to the structure and some specific details of the manuscript.

- The first two paragraphs of the introduction are lengthy and have too little focus on the actual topic. A more relevant introduction seems to be hiding in the first paragraph of the results section.

***Response****: We thank the reviewer for the comment, and have rewritten the background section to make it more relevant and comply with other comments.*

- I would recommend replacing the term enhancer with "regulatory sequence"/"sequence" throughout the manuscript. The definition of the term enhancer is somewhat unclear and introduces an unnecessary and artificial limitation of MPRAs and this method.

***Response****: We have corrected the text accordingly. Sequences tested in an MPRA are now referred to as ‘sequences’.*

- p5, l28-29: barcode-level effects on RNA; I do not agree with this statement. Barcodes could easily influence UTR stability and RNA decay, thereby affecting transcriptional activity. Further, (and similar to the DNA) the barcode will also effect amplification efficiencies in library prep.

***Response****: We thank the reviewer for pointing this out, and agree with the comment. Our intention was not to suggest that barcodes don’t affect the RNA counts, but simply that when modelling the data, one would usually want to extract a single alpha estimate across barcodes, as opposed to a separate estimate for each barcode. A separate estimate for each barcode would make for a more accurate model, but is ultimately less useful. We rephrased this portion of the text to be clearer about why we recommend not including barcode-effects in the RNA model, and explicitly recognize that barcode-specific effects may impact RNA counts as well.*

- p7/Fig.2 & 3: I am wondering whether labeling and description of agg.ratio/mean ratio is consistent throughout the figures and the text. It was unexpected for me that, the mean ratio method is supposed to have the lower variance, while being described earlier as most sensitive to noise. I would ask that a short description of agg. ratio/mean ratio is included in the figure legends, for example the formulas given in the methods.

***Response****: We appreciate the comment and understand the confusion. We indeed had two issues with the agg.ratio and mean ratio estimates: (1) the legend in figure 3A had wrong labels, and (2) the analysis underlying figure 2A was mistakenly using zero-padded data (due to a bug in the code), which inflated the variance of the aggregated estimates. Both issues have been corrected, with minor changes to the interpretation (namely, the aggregated ratio is consistently more robust than the mean ratio).*

*Additionally, we added the formulas for calculating both estimates to the legend of Figure 2, as well as the main text.*

- p11, l15: correct citation

- p 11,l16: introduce BH/Benjamini-Hochberg abbreviation at first mention

- p 11,l22: fix incorrect character after "Pearson's r"

***Response****: We have now corrected the text accordingly.*

-p 11,l46ff/Supp. Fig 7: analysis seems over the top for this purpose. Consider removing/moving to the supplement.

***Response****: We thank the reviewer for this comment, however to comply with the editorial and other reviewer’s comments we have expanded this section and provided further analysis to substantiate MPRAnalyze’s utility.*

- legend Figure 1: "plots for both libraries" -> "plots for DNA and RNA libraries"

***Response****: We thank the reviewer for pointing this out, and corrected the text accordingly.*

**Second round of review**

**Reviewer 1**

**Comment to author**

The authors have addressed most of the issues raised in the previous review (with a couple exceptions, noted below). The new introduction is much better, and the new analyses strengthen the paper by providing examples of the benefits from using MPRAnalyze.

Despite these improvements, in its current form the paper is better suited to a specialist statistical journal. When given the option, the authors choose to emphasize statistical features (such as p-value control) over biological insights that can be derived from using this method. The text is at times repetitive and lacks some important highlevel sections to help orient the reader. Some more detailed suggestions follow, but these are problems throughout the manuscript and it needs substantial revisions to improve the flow and organization.

It is also worth noting that the Supplemental File 5 describing more detailed methods was not provided, so I was unable to comment on it.

Suggestions

The introduction primarily cites older papers, which is not suitable for a technology that is as fast-moving as MPRAs. For example, citation 4 is from 2009 and ignores more recent efforts such as [1] (which even shares an author with this study!).

***Response****: We thank the reviewer for pointing this out and have added more recent citations to the introduction.*

The introduction also lacks any citation for the MPRA technology. This should be rectified by citing relevant primary literature and/or relevant reviews (such as [2,3]). The first paragraph of the Results section also introduces MPRAs without citation.

***Response****: We thank the reviewer and have added appropriate citations to both sections.*

The authors use a variety of terms to describe different types of MPRA experiments. They should make sure that these match the accepted terms in the field by consulting a current reference such as [3].

***Response****: We thank the reviewer for pointing this out. Since MPRAs are a developing field, various terms are used in different studies, and we made an effort to use common terms that are easily understood, and we did not find any contradicting terms used in the provided reference.*

When mentioning DESeq2 for the first time at the end of the introduction, the authors should state that it is typically used for RNA-seq.

***Response****: We thank the reviewer and have added this statement to the text.*

It is unclear what the second part of the first Results paragraph (about multiple barcodes) is motivating. This section should be re-worked so that it’s tied directly to the MPRAnalyze method.

***Response****: We thank the reviewer for pointing this out, and have added an explicit motivation to the end of this paragraph, directly connecting it to the presentation of MPRAnalyze in the following section.*

The MPRAnalyze Model section includes a lot of mathematical details that would be better placed in the Methods. Instead, this part of the manuscript should include an explanation of the method and its motivation in plain language with no equations that is accessible to researchers with a minimal background in statistics who might want to use the output of the method.

***Response****: We appreciate this comment by the reviewer, and have made changes to the text to make the method description more approachable to readers with minimal background.*

The authors cite DESeq2 for the use of the negative binomial, but this is not the correct citation. I provided two citations in my previous review that significantly predate this one that should be used instead: [4,5].

***Response****: We thank the reviewer for pointing these out, and have replaced the DESeq2 citation to citing the edgeR paper.*

The comparison sections (3-6) would benefit from an introductory paragraph that lists the types of analyses that will be shown to help orient the reader. This would also be a good place to detail why certain competing methods are used in some comparisons and not others – as well as describing why MPRAnalyze is so useful because it can do all of these things. It might be better to turn this into a single “Performance” section with sub-headings for each type of analysis.

***Response****: We thank the reviewer for the comment, and have added introductory statements to each comparison section to summarize the analyses performed and comparisons made.*

The Classification section is split into three parts and is particularly long and repetitive. The authors should consider streamlining it into a single section. This is applicable to other analysis sections as well.

***Response****: We appreciate this feedback from the reviewer. The section was divided to multiple subsection to make it easier to follow, since the section makes multiple points and therefore does not easily follow a linear narrative. We have edited the text to avoid making repetitive statements and make the text overall more concise.*

In the Classification section, the authors refer to “limitations of the summary statistics demonstrated above” but it would be helpful to have a brief explanation of how these limitations impair this particular type of MPRA analysis.

***Response****: We appreciate the reviewer suggestion, and have added a brief explanation to this paragraph as suggested.*

The description of using MAD to compare alpha balues to the null should be moved to the methods.

***Response****: We thank the reviewer for this suggestion, however the details of how the classification hypothesis testing is performed are of interest to many of the potential users of MPRAnalyze (based on conversations with potential and active users of the package), and we therefore opted to describe it in the main text. We have edited the explanation to make it more concise.*

The “Caveats of using methods designed for differential expressoin” section can be trimmed down a lot, since the conclusion is already stated at the start of the section.

***Response****: We thank the reviewer for this comment. In the revised text, the analysis is mentioned in the beginning of the section but results are not discussed before this subsection, avoiding making this subsection repetitive. Additionally, the text for this subsection was shortened.*

For the “Comparative Studies” section, the authors use the Inoue-Kreimer dataset only. There should be some explanation of why this dataset was chosen over the others. Were the other datasets unusable?

***Response****: We appreciate the reviewer pointing out this confusion. The Inoue-Kreimer dataset is the only comparative benchmark dataset we included in the paper, as noted in Table 1.*

In the “Comparative Studies” section the authors seemt to use the number of significant results as a performance metric. This is clearly flawed because more results is not the same as more correct or validated results. The paragraph that starts with discussion of TEAD2 and NRF1 is much more compelling than the discussion of p-values that the authors choose to begin with. I recommend that the authors restructure the various result sections to bring biologically relevant findings forward.

***Response****: We thank the reviewer for this comment and agree that more results are not necessarily better. For this reason, we included in our analysis the “consensus + noise” option, which demonstrates the enrichment analysis if the consensus results were padded with random results to match MPRAnalyze’s numbers. This clearly demonstrates that MPRAnalyze’s additional results are strengthening the biological signal in the data, and are not simple inflation due to additional results.*

The authors should be precise when describing sequences and not use bare gene names as a substitute for transcription factor binding sites. For example, they did not find “two of the core pluripotency factors”, they found “binding sites for two of the core pluripotency factors”. This is an issue throughout the section.

***Response****: We thank the reviewer for pointing this out and have edited the text to accurately describe the nature of our results.*

“Detecting temporal activity” describes a major advantage that MPRAnalyze has over other tools. This should be brought forward and highlighted.

***Response****: We thank the reviewer for this suggestion, this is now mentioned in the beginning of the comparative analysis section.*

I wasn’t clear on why the authors clustered the temporal sequences. This needs more motivation and exploration.

***Response****: The temporal sequences were clustered to group them by temporal pattern, so we can analyze early-active and lateactive sequences separately. This point has been clarified in the text.*

In the “Allelic Comparison” section, the authors presume that HepG2’s high correlation is “due to the higher number of replicates in the HepG2 data” but they could test this by subsampling.

***Response****: We thank the reviewer for suggesting this, and have now included the subsampling analysis which validates our hypothesis (Figure S11).*

For me, the cell-line specific effects was one of the strongest demonstrations in the paper and it’s buried as the last result. I suggest the authors find a way to bring this forward in the manuscript and highlight it.

***Response****: We thank the reviewer for this suggestion, this result is now mentioned in the beginning of the allelic comparison analysis section.*

In the Discussion, the authors describe MPRAs as “a relatively nascent technology”. Perhaps “actively developed technology” or “fast-moving field” would be better choices, as nascent implies that it’s not ready for serious use.

***Response****: We appreciate the comment and have edited the text accordingly.*

The authors point out in the discussion that MPRAnalyze benefits from larger experiments with more barcodes. It’s worth noting that experiments are getting bigger every year, and so MPRAnalyze is poised to scale with them.

***Response****: We appreciate the reviewer pointing this out and agree that this seems to be the general direction of the field, and we now cite recent examples that demonstrate these recommendations.*

Figures 4B, 5C-I seem to suffer from significant overplotting. Using a hexbin density or points with an alpha-value could help with this.

***Response****: We appreciate the comment and have replotted these figures with a hexbin density plot.*

I’m not sure what the point of Figure 5J is. Why not just make 5K-L a bit bigger?

***Response****: We appreciate the suggestion by the reviewer. Figure 5J represents a graphical representation of the enrichment analysis we performed, meant to clarify the enrichment analysis in a visual and intuitive manner since a textual explanation can be difficult to follow.*

1. Chatterjee S, Ahituv N. Gene Regulatory Elements, Major Drivers of Human Disease. Annu Rev Genomics Hum Genet. 2017;18:45–63.

2. Inoue F, Ahituv N. Decoding enhancers using massively parallel reporter assays. Genomics. 2015;106:159–64.

3. Kinney JB, McCandlish DM. Massively Parallel Assays and Quantitative Sequence–Function Relationships. Annu Rev Genomics Hum Genet. 2019;20:null.

4. Lönnstedt I, Speed T. REPLICATED MICROARRAY DATA. Stat Sin. 2002;12:31–46.

5. Robinson MD, McCarthy DJ, Smyth GK. edgeR: a Bioconductor package for differential expression analysis of digital gene expression data. Bioinformatics. 2010;26:139–40.

**Reviewer 2**

The authors have addressed all my concerns and comments

**Reviewer 3**

In their revised manuscript "MPRAnalyze - A statistical framework for Massively Parallel Reporter Assays", Tal Ashuach et al. addressed all my prior comments with this major revision. In response to other reviewer comments, they have extended their analyses, but also improved the flow of the manuscript. I found minor textual errors and problems with figure quality (only in the version created by the journal from the TeX submission), but otherwise do not have any further comments. I recommend accepting the manuscript.

Formatting/text errors:

- Spaces missing between author first/last names

- "non-coding" rather than "non coding"?

- consistent usage of alpha vs. \alpha?

- "quadratic relationship between -between- the mean and the variance,"

- "Since -T-[t]hese analyses are limited by"

- "being comparably sensitive to the other-h- methods"

- "Mpralm -enables using-[can use] either the aggregated ratio or the mean ratio as the statistic"

- "Spearman correlation *>* 0.63" [problems with character formatting]

- "Complex experimental design[s] can include interaction studies,"

- "variosu" -> "various" - "MPRA datasets makes negative control[s] a valuable"

- "Negative control sequences we[re] generated by scrambling"

- "Each sequence-s- was associated with 90"

- "allelic comparisons, while the package vignette us-ing-[es] the sum across"

- "An implementation of this simulation process i-n-s included"

- "(- -batch"

*- section "Differential Deletions Analysis": mention that this about the Mattioli data set*

***Response****: We thank the reviewer for pointing these out to us and have corrected all of these in the text. With regard to consistent \alpha vs. alpha use, we now refer to these values as the mathematical symbol (\alpha) in the text, however for readability they are still referred to as ‘alpha’ in some figures.*
